# On the characteristic equation $\lambda =\alpha _{1}+(\alpha _{2}+\alpha _{3}\lambda )e^{-\lambda }$ and its use in the context of a cell population model

**DOI:** 10.1007/s00285-015-0918-8

**Published:** 2015-08-06

**Authors:** Odo Diekmann, Philipp Getto, Yukihiko Nakata

**Affiliations:** Mathematical Institute, University of Utrecht, Utrecht, The Netherlands; TU Dresden, Fachrichtung Mathematik, Institut für Analysis, 01062 Dresden, Germany; Graduate School of Mathematical Sciences, University of Tokyo, 3-8-1 Komaba, Meguro-ku, Tokyo, 153-8914 Japan

**Keywords:** 34K20, 37N25, 45D05, 65L03, 92D25

## Abstract

In this paper we characterize the stability boundary in the $(\alpha _{1},\alpha _{2})$-plane, for fixed $\alpha _{3}$ with $-1<\alpha _{3}<+1$, for the characteristic equation from the title. Subsequently we describe a nonlinear cell population model involving quiescence and show that this characteristic equation governs the (in)stability of the nontrivial steady state. By relating the parameters of the cell model to the $\alpha _{i}$, we are able to derive some biological conclusions.

**Electronic supplementary material:**

The online version of this article (doi:10.1007/s00285-015-0918-8) contains supplementary material, which is available to authorized users.

## Introduction

The characteristic equation

1.1 $$\begin{aligned} \lambda =\alpha _{1}+\alpha _{2}e^{-\lambda } \end{aligned}$$

for $\lambda \in {\mathbb {C}}$, with parameters $\alpha _{1},\alpha _{2}\in {\mathbb {R}}$, corresponds to the delay differential equation

1.2 $$\begin{aligned} {\dot{x}}(t)=\alpha _{1}x(t)+\alpha _{2}x(t-1). \end{aligned}$$

The fact that (1.1) can have, for $\alpha _{1},\alpha _{2}<0$, solutions with $\text {Re}\lambda >0$ therefore leads to the dictum that delayed negative feedback can lead to oscillatory instability.

**Fig. 1:**
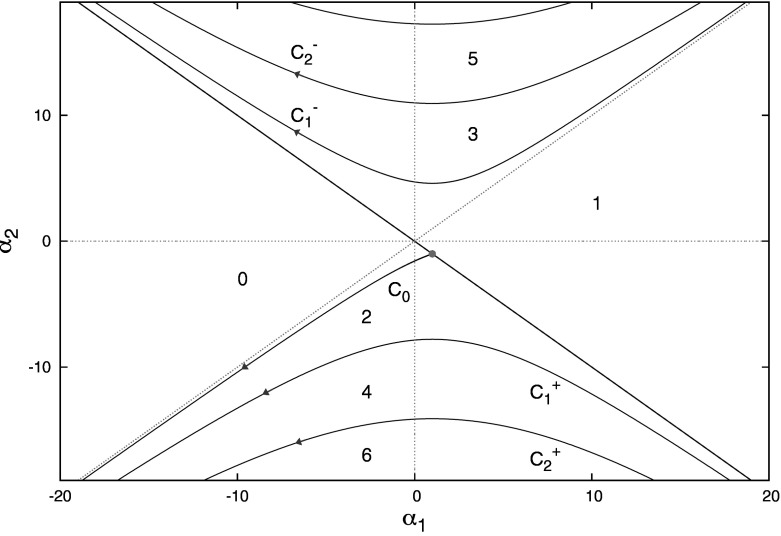
The numbers specify the number of roots of (1.1) with $\text {Re}\lambda >0$ for $(\alpha _{1},\alpha _{2})$ in the corresponding region of the parameter plane

Equation (1.1) can be analyzed in great detail (see e.g. Section III.3 in Èl’sgol’ts and Norkin 1973; Hayes 1950; Chapter 13.7 in Bellman and Cooke 1963; Chapter XI in Diekmann et al. 1991). The outcome can be conveniently summarized in the diagram depicted in Fig. 1 (corresponding to what Èl’sgol’ts and Norkin 1973 call the method of D-partitions; also see Breda 2012).

In the context of specific models, (1.2) usually arises by linearization of a nonlinear equation around a steady state. In such a situation $\alpha _{1},\alpha _{2}$ are (sometimes rather complicated) functions of the original model parameters. As emphasized in Chapter XI in Diekmann et al. (1991) and the recent didactical note (Diekmann and Korvasova 2013), one can combine the detailed knowledge embodied in Fig. 1 with an analysis of the parameter map, that relates the original parameters to $(\alpha _{1},\alpha _{2})$, in order to obtain stability and bifurcation results for the steady state of the nonlinear equation. Even if the aim is to perform a one-parameter Hopf bifurcation study, it is much more efficient to derive first the two parameter picture of Fig. 1, see Diekmann and Korvasova (2013). We also refer to Insperger and Stépán (2011), Kuang (1993), Michiels and Niculescu (2014), Stépán (1989) for a systematic approach to deriving stability conditions for steady states of delay differential equations via an analysis of characteristic equations and to Cheng and Lin (2009) for potentially useful general theory.

In Sect. 3 we shall formulate a model for a population of cells that can either be on the way to division or quiescent. The model is very close in spirit to the one formulated and studied by Gyllenberg and Webb in their pioneering paper (Gyllenberg and Webb 1990). But our formulation is in terms of delay equations, more precisely a system consisting of one Renewal Equation (RE) and one Delay Differential Equation (DDE) (Diekmann and Gyllenberg 2012; Diekmann et al. 2010; 2007/2008), rather than in terms of PDE as in Gyllenberg and Webb (1990). The delay equation formulation enables a relatively painless derivation of the characteristic equation whose roots govern the (in)stability of the (unique) nontrivial steady state. In the relatively simple case considered here (see Alarcón et al. 2014; Borges et al. 2014 for a more general model formulation), the RE degenerates into a difference equation in continuous time! The characteristic equation corresponding to the linearization about the nontrivial steady state takes the form

1.3 $$\begin{aligned} \lambda =\alpha _{1}+\left( \alpha _{2}+\alpha _{3}\lambda \right) e^{-\lambda }. \end{aligned}$$

The goal of the present paper is to investigate how the picture of Fig. 1 deforms if we let $\alpha _{3}$ grow away from zero (but restrict to $-1<\alpha _{3}<+1$, for reasons explained around Lemma 2.2 below). See the supplementary material for a movie showing how the curves depicted in Fig. 1 move in the $(\alpha _{1},\alpha _{2})$ plane when $\alpha _{3}$ ranges from $-1$ to $+1$. Our main conclusion is that, from a qualitative point of view, nothing changes at all.

## The stability region $S(\alpha _{3})$ in the $(\alpha _{1},\alpha _{2})$-plane

Our aim is to characterize

2.1 $$\begin{aligned} S(\alpha _{3})=\left\{ \left( \alpha _{1},\alpha _{2}\right) : (1.3) \text { has no roots }\lambda \text { with } \text {Re}\lambda \ge 0\right\} \end{aligned}$$

by a precise description of its boundary. We shall show in Theorem 2.1 that this boundary consists of the half-line

2.2 $$\begin{aligned} L(\alpha _{3})=\left\{ \left( \alpha _{1},\alpha _{2}\right) : \alpha _{2}=-\alpha _{1},\ \alpha _{1}\le 1-\alpha _{3}\right\} , \end{aligned}$$

corresponding to $\lambda =0$ being a root of (1.3), and the curve

2.3 $$\begin{aligned} C_{0}(\alpha _{3})=\left\{ \left( c_{1}\left( \omega , \alpha _{3}\right) ,c_{2}\left( \omega ,\alpha _{3}\right) \right) :0\le \omega <\pi \right\} \end{aligned}$$

where

2.4a $$\begin{aligned} c_{1}(\omega ,\alpha _{3})&=\frac{\omega }{\sin \omega }\left( \cos \omega -\alpha _{3}\right) , \end{aligned}$$

**Fig. 2:**
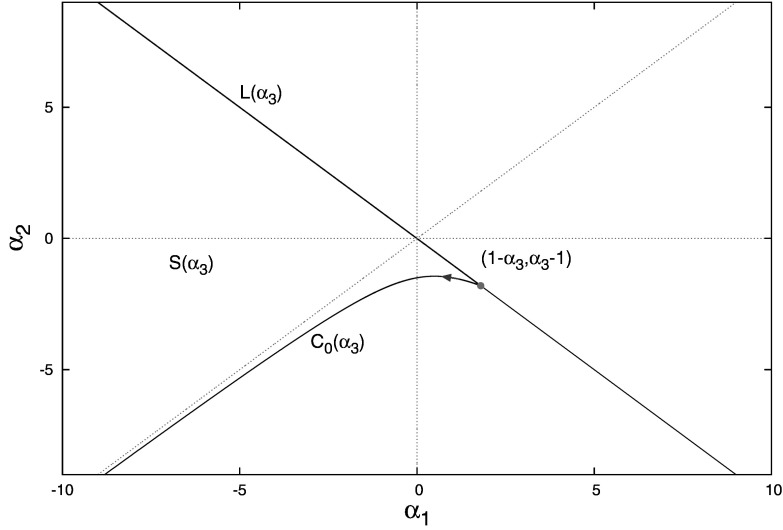
Stability region $S(\alpha _{3})$ for the characteristic equation (1.3). The boundary of $S(\alpha _{3})$ consists of the half-line $L(\alpha _{3})$ and the curve $C_{0}(\alpha _{3})$

2.4b $$\begin{aligned} c_{2}(\omega ,\alpha _{3})&=\frac{\omega }{\sin \omega }\left( \alpha _{3}\cos \omega -1\right) , \end{aligned}$$

corresponding to $\lambda =\pm i\omega ,\ 0\le \omega <\pi$, being a root of (1.3). Note that $L(\alpha _{3})$ and $C_{0}(\alpha _{3})$ intersect in $(1-\alpha _{3},\alpha _{3}-1)$, corresponding to $\lambda =0$ being a double root, see Lemma 2.4 below. The stability region $S(\alpha _3)$ is depicted in Fig. 2.

### **Theorem 2.1**

Let $-1<\alpha _{3}<1$. $S(\alpha _{3})$ is the connected open subset of ${\mathbb {R}}^{2}$ that contains $\{ (\alpha _{1},0):\alpha _{1}<0\}$ and has $L(\alpha _{3})\cup C_{0}(\alpha _{3})$ as its boundary.

The proof has quite a few components, so we build it up gradually by deriving auxiliary results. Our first step is to exclude that roots can enter the right half plane at $\lambda =\infty$ when $\alpha _{1}$ and $\alpha _{2}$ are varied. This leads to the constraint $-1<\alpha _{3}<+1$.

### **Lemma 2.2**

Let $|\alpha _{3}|<1$ and let $\lambda$ be a root of (1.3) with $\text {Re}\lambda \ge 0$. Then

2.5 $$\begin{aligned} \left| \lambda \right| \le \frac{\left| \alpha _{1}\right| + \left| \alpha _{2}\right| }{1-\left| \alpha _{3} \right| }. \end{aligned}$$

### *Proof*

We write (1.3) in the form

$$\begin{aligned} \lambda \left( 1-\alpha _{3}e^{-\lambda }\right) =\alpha _{1}+ \alpha _{2}e^{-\lambda } \end{aligned}$$

and take absolute values at both sides. Using

$$\begin{aligned} \left| \alpha _{1}+\alpha _{2}e^{-\lambda }\right|&\le \left| \alpha _{1}\right| +\left| \alpha _{2}\right| e^{-\text {Re}\lambda }\le \left| \alpha _{1}\right| +\left| \alpha _{2}\right| \nonumber \\ \left| \alpha _{3}e^{-\lambda }\right|&=\left| \alpha _{3}\right| e^{-\text {Re}\lambda }\le \left| \alpha _{3}\right| <1\nonumber \\ \left| 1-z\right|&\ge 1-\left| z\right| >0\text { when }\left| z\right| <1 \end{aligned}$$

we obtain the estimate (2.5). $\square$

To further illustrate the importance of the restriction $|\alpha _{3}|<1$, we formulate a corollary of Theorem 1.1 in Chapter 3 of Kuang (1993):

### **Lemma 2.3**

When $|\alpha _{3}|>1$, Eq. (1.3) has infinitely many roots in the right half plane $\text {Re}\lambda >0$.

We refer to Lemma 2.12 below for a description of the limiting behaviour for $\alpha _{3}\uparrow 1$ and $\alpha _{3}\downarrow -1$. In order to facilitate various formulations, we adopt the convention that in the rest of this section the inequalities

2.6 $$\begin{aligned} -1<\alpha _{3}<1 \end{aligned}$$

hold, unless stated otherwise.

Lemma 2.2 implies (by way of Rouché’s Theorem, see Lemma 2.8 in Chapter XI of Diekmann et al. 1991) that at the boundary of $S(\alpha _{3})$ the Eq. (1.3) has either a root $\lambda =0$ or a pair of roots $\lambda =\pm i\omega ,\ \omega >0$.

### **Lemma 2.4**

1. $\lambda =0$ is a root of (1.3) iff $\alpha _{1}+\alpha _{2}=0$. It is a double root iff in addition $\alpha _{2}=\alpha _{3}-1$ (and a triple root iff in addition $\alpha _{3}=-1$).
2. $\lambda =\pm i\omega ,\ \omega >0$ is a complex conjugate pair of roots of (1.3) iff
  $$\begin{aligned} \left( \alpha _{1},\alpha _{2}\right) =\left( c_{1}\left( \omega , \alpha _{3}\right) ,c_{2}\left( \omega ,\alpha _{3}\right) \right) \end{aligned}$$
  with $c_{i}$ defined by (2.4).
3. $$\lim _{\omega \rightarrow 0}(c_{1}(\omega ,\alpha _{3}), c_{2}(\omega ,\alpha _{3}))=(1-\alpha _{3}, \alpha _{3}-1)$$.

### *Proof*

1. Substituting $\lambda =0$ into (1.3) we obtain $0=\alpha _{1}+\alpha _{2}$. Taylor expanding $e^{-\lambda }$ we can write (1.3) in the form
  $$\begin{aligned} \lambda =\alpha _{1}+\alpha _{2}+\left( \alpha _{3}- \alpha _{2}\right) \lambda +\left( \frac{\alpha _{2}}{2}-\alpha _{3} \right) \lambda ^{2}+O(\lambda ^{3}) \end{aligned}$$
  and conclude that for $\alpha _{1}+\alpha _{2}=0$ the root $\lambda =0$ is a double root iff $\alpha _{2}=\alpha _{3}-1$ and a triple root iff in addition $\alpha _{3}=-1$.
2. In terms of real variables $\mu$ and $\omega$ such that
  2.7 $$\begin{aligned} \lambda =\mu +i\omega \end{aligned}$$
  the complex equation (1.3) amounts to the two real equations $G_{i}=0,\ i=1,2$, where $G_{1}$ corresponds to the real part and is given by
  2.8 $$\begin{aligned} G_{1}(\alpha _{1},\alpha _{2},\mu ,\omega )=-\mu +\alpha _{1}+ \left( \alpha _{2}+\alpha _{3}\mu \right) \cos \omega e^{-\mu }+ \alpha _{3}\omega \sin \omega e^{-\mu } \end{aligned}$$
  while $G_{2}$ corresponds to the imaginary part and is given by
  2.9 $$\begin{aligned} G_{2}(\alpha _{1},\alpha _{2},\mu ,\omega )=-\omega - \left( \alpha _{2}+\alpha _{3}\mu \right) \sin \omega e^{-\mu }+ \alpha _{3}\omega \cos \omega e^{-\mu }. \end{aligned}$$
  For $\mu =0$ the equations reduce to
  $$\begin{aligned} \alpha _{1}+\alpha _{2}\cos \omega +\alpha _{3}\omega \sin \omega&=0,\\ -\omega -\alpha _{2}\sin \omega +\alpha _{3}\omega \cos \omega&=0. \end{aligned}$$
  Solving the second for $\alpha _{2}$ we obtain $\alpha _{2}=c_{2}(\omega ,\alpha _{3})$. Next we can solve the first for $\alpha _{1}$. This yields $\alpha _{1}=c_{1}(\omega ,\alpha _{3})$.
3. Since $\lim _{\omega \rightarrow 0}\frac{\omega }{\sin \omega }=1$ and $\cos 0=1$, it is clear that $c_{1}(\omega ,\alpha _{3})\rightarrow 1-\alpha _{3}$ and $c_{2}(\omega ,\alpha _{3})\rightarrow \alpha _{3}-1$ for $\omega \rightarrow 0$.$\square$

Our next step is to identify for any $\alpha _{3}$ at least one point in the $(\alpha _{1},\alpha _{2})$-plane that belongs to $S(\alpha _{3})$. Clearly Eq. (1.3) is very simple if $(\alpha _{1},\alpha _{2})=(0,0)$, but this point lies on $L(\alpha _{3})$. The idea is to focus on a neighborhood, i.e., first consider $(\alpha _{1},\alpha _{2})=(0,0)$ and next perturb.

### **Lemma 2.5**

1. For $(\alpha _{1},\alpha _{2})=(0,0)$ the roots of (1.3) are given by $\lambda =0$ and, if $\alpha _{3}\not =0$,
  $$\begin{aligned} \lambda&=\ln \alpha _{3}+2k\pi i,\ k\in {\mathbb {Z}},\ \text {if }\alpha _{3}>0,\\ \lambda&=\ln \left( -\alpha _{3}\right) +\left( 2k+1\right) \pi i,\ k\in {\mathbb {Z}},\ \text { if }\alpha _{3}<0, \end{aligned}$$
  while for $\alpha _{3}=0$, $\lambda =0$ is the one and only root.
2. Consider $\alpha _{2}=0$ and $|\alpha _{1}|$ small. The only root of (1.3) that can possibly lie in the right half plane is given by
  $$\begin{aligned} \lambda =\frac{\alpha _{1}}{1-\alpha _{3}}+O(\alpha _{1}^{2}). \end{aligned}$$
  As a consequence, the points $(\alpha _{1},0)$ with $\alpha _{1}<0$, $|\alpha _{1}|$ small, belong to $S(\alpha _{3})$.

### *Proof*

1. For $(\alpha _{1},\alpha _{2})=(0,0)$ Eq. (1.3) reduces to $\lambda =\alpha _{3}\lambda e^{-\lambda }$. So $\lambda =0$ is a root and all other roots satisfy $1=\alpha _{3}e^{-\lambda }$.
2. So for $(\alpha _{1},\alpha _{2})=(0,0)$ we have one root $\lambda =0$ on the imaginary axis and, if $\alpha _{3}\not =0$, countably many other roots that are at a uniformly positive distance away from the imaginary axis in the left half plane. According to the Implicit Function Theorem, there exists for small $\alpha _{1}$ a root
  $$\begin{aligned} \lambda =\frac{\alpha _{1}}{1-\alpha _{3}}+O(\alpha _{1}^{2}) \end{aligned}$$
  if we keep $\alpha _{2}=0$, and this is the only root in a small ball *B* around $\lambda =0$. According to a variant of Rouché’s Theorem, see Lemma 2.8 of Chapter XI of Diekmann et al. (1991), there are for small $\alpha _{1}$ no roots in the open set $\{ \lambda :\text {Re}\lambda >0,\ \lambda \not \in \overline{B}\} .$ (Note that here we use Lemma 2.2 to compensate for the non-compactness of the closure of this set.) $\square$

As we saw in Part 2 of the lemma above, it is helpful to know how the root $\lambda =0$ moves in ${\mathbb {C}}$ if we move $(\alpha _{1},\alpha _{2})$ away from the line $\alpha _{1}+\alpha _{2}=0$. The following result shows that if we cross $L(\alpha _{3})$ transversally, away from its endpoint, from below to above, a real root of (1.3) crosses the imaginary axis from left to right.

### **Lemma 2.6**

Suppose that $[-\overline{\varepsilon },\overline{\varepsilon }]\rightarrow {\mathbb {R}}^{2};\ \varepsilon \mapsto (\alpha _{1},\alpha _{2})(\varepsilon )$, for some $\overline{\varepsilon }>0$, is $C^{1}$ with $(\alpha _{1},\alpha _{2})(0)=(\gamma ,-\gamma )$ for some $\gamma \not =1-\alpha _{3}$. Then (1.3), for small $\varepsilon$, has a real root $\lambda$, which is of the form

$$\begin{aligned} \lambda =\frac{\alpha _{1}^{\prime }(0)+\alpha _{2}^{\prime } (0)}{1-\alpha _{3}-\gamma }\varepsilon +O(\varepsilon ^{2}). \end{aligned}$$

If additionally $(\alpha _{1},\alpha _{2})^{\prime }(0)(1,1)^{t}>0$ and $\gamma <1-\alpha _{3}$, then $\lambda <0$ for small negative $\varepsilon$ and $\lambda >0$ for small positive $\varepsilon$.

We omit the elementary proof.

In a similar spirit, we want to know how the roots $\lambda =\pm i\omega$ move in ${\mathbb {C}}$ if we move $(\alpha _{1},\alpha _{2})$ away from the curve $C_{0}(\alpha _{3})$. In Section XI.2 of Diekmann et al. (1991) it is explained that the crucial quantity is the sign of $\text {det}M$, where

$$\begin{aligned} M=\left. \left( \begin{array}{c@{\quad }c} \frac{\partial G_{1}}{\partial \alpha _{1}} &{} \frac{\partial G_{1}}{\partial \alpha _{2}}\\ \frac{\partial G_{2}}{\partial \alpha _{1}} &{} \frac{\partial G_{2}}{\partial \alpha _{2}} \end{array}\right) \right| _{\left( \alpha _{1},\alpha _{2},0,\omega \right) } \end{aligned}$$

with $G_{1}$ defined by (2.8) and $G_{2}$ by (2.9). So in the present case we have

$$\begin{aligned} M=\left( \begin{array}{c@{\quad }c} 1 &{} \cos \omega \\ 0 &{} -\sin \omega \end{array}\right) \end{aligned}$$

and $\text {det}M=-\sin \omega <0$ for $0<\omega <\pi$. In order to define what we mean by “to the left” and “to the right”, we provide $C_{0}(\alpha _{3})$ with the orientation of increasing $\omega$. According to Proposition 2.13 in Chapter XI of Diekmann et al. (1991), we can draw the following conclusion.

### **Lemma 2.7**

When we cross $C_{0}(\alpha _{3})$ from right to left at a point corresponding to $\omega >0$, a pair of complex conjugate roots of (1.3) crosses the imaginary axis from left to right.

Motivated by Lemma 2.4(2) we define for $k=1,2,\dots$ the intervals

2.10a $$\begin{aligned} I_{k}^{-}&=\left( \left( 2k-1\right) \pi ,2k\pi \right) \end{aligned}$$

2.10b $$\begin{aligned} I_{k}^{+}&=\left( 2k\pi ,\left( 2k+1\right) \pi \right) \end{aligned}$$

and the curves

2.11 $$\begin{aligned} C_{k}^{\pm }(\alpha _{3})=\left\{ \left( c_{1}\left( \omega , \alpha _{3}\right) ,c_{2}\left( \omega ,\alpha _{3}\right) \right) : \omega \in I_{k}^{\pm }\right\} \end{aligned}$$

with $c_{i}$ defined by (2.4). In the next lemma we collect some observations that help to understand the shape of the curves $C_{0},\ C_{k}^{\pm }$.

### **Lemma 2.8**

For $c_{i}(\omega ,\alpha _{3}),\ i=1,2$ defined by (2.4) we have

1. $$\frac{\partial c_{1}}{\partial \omega }(\omega ,\alpha _{3})<0$$ for $$\omega >0,\ \omega \not =m\pi ,\ m=1,2,\dots ,$$
2. $$c_{1}(\omega ,\alpha _{3})\rightarrow {\left\{ \begin{array}{ll} -\infty &{} \text {as }\omega \uparrow m\pi \\ +\infty &{} \text {as }\omega \downarrow m\pi \end{array}\right. },\ m=1,2,\dots ,$$
3. $$c_{2}(\omega ,\alpha _{3}){\left\{ \begin{array}{ll} >0 &{} \text {on }I_{k}^{-}\\ <0 &{} \text {on }[0,\pi )\text { and on }I_{k}^{+} \end{array}\right. },$$
4. $\frac{c_{1}}{c_{2}}(\cdot ,\alpha _{3})$ is periodic with period $2\pi$ and decreases from $+1$ to $-1$ on $I_{k}^{-}$ and increases from $-1$ to $+1$ on $[0,\pi )$*and on*$I_{k}^{+}$,
5. $$\frac{c_{1}}{c_{2}}(\omega +\pi ,\alpha _{3})=- \frac{c_{1}}{c_{2}}(\omega ,-\alpha _{3}).$$

### *Proof*

1. $$\begin{aligned} \frac{\partial c_{1}}{\partial \omega }\left( \omega ,\alpha _{3}\right) = \frac{\sin \omega \cos \omega -\omega +\alpha _{3}\left( \omega \cos \omega - \sin \omega \right) }{\sin ^{2}\omega }. \end{aligned}$$
  Now note that
  $$\begin{aligned} \frac{\partial c_{1}}{\partial \omega }\left( \omega ,-1\right) = \frac{\left( \cos \omega +1\right) \left( \sin \omega -\omega \right) }{ \sin ^{2}\omega }<0\quad \text {for }\omega >0,\ \omega \not =m\pi \end{aligned}$$
  and that
  $$\begin{aligned} \frac{\partial c_{1}}{\partial \omega }\left( \omega ,+1\right) = \frac{\left( \cos \omega -1\right) \left( \sin \omega +\omega \right) }{ \sin ^{2}\omega }<0\quad \text {for }\omega >0,\ \omega \not =m\pi . \end{aligned}$$
  Since $\frac{\partial c_{1}}{\partial \omega }$ is a linear function of $\alpha _{3}$, it follows that for $-1<\alpha _{3}<+1$ the inequality
  $$\begin{aligned} \frac{\partial c_{1}}{\partial \omega }\left( \omega ,\alpha _{3}\right) <0 \end{aligned}$$
  holds for $$\omega >0,\ \omega \not =m\pi $$.
2. At $\omega =m\pi$ with *m* odd, $\sin \omega$ switches from positive to negative values and $\cos \omega -\alpha _{3}=-1-\alpha _{3}<0$. For even *m*, $\sin \omega$ switches from negative to positive values, but $\cos \omega -\alpha _{3}=1-\alpha _{3}>0$.
3. Since $\alpha _{3}\cos \omega -1<0$, the sign of $c_{2}$ is the opposite of the sign of $\sin \omega$.
4. $$\begin{aligned} \frac{c_{1}}{c_{2}}\left( \omega ,\alpha _{3}\right) = \frac{\cos \omega -\alpha _{3}}{\alpha _{3}\cos \omega -1} \end{aligned}$$
  is obviously $2\pi$-periodic.
  $$\begin{aligned} \frac{d}{d\omega }\frac{c_{1}}{c_{2}}\left( \omega , \alpha _{3}\right) =\frac{\sin \omega \left( 1-\alpha _{3}^{2} \right) }{\left( \alpha _{3}\cos \omega -1\right) ^{2}}, \end{aligned}$$
  so $\frac{c_{1}}{c_{2}}$ is an increasing function of $\omega$ on intervals on which $\sin \omega \!>\!0$ and a decreasing function of $\omega$ on intervals on which $\sin \omega <0$. Moreover
  $$\begin{aligned} \frac{c_{1}}{c_{2}}\left( m\pi ,\alpha _{3}\right) = \frac{\left( -1\right) ^{m}-\alpha _{3}}{\left( -1\right) ^{m} \alpha _{3}-1}=\left( -1\right) ^{m+1}. \end{aligned}$$
5. $$\begin{aligned} \frac{c_{1}}{c_{2}}(\omega +\pi ,\alpha _{3})&= \frac{\cos \left( \omega +\pi \right) -\alpha _{3}}{\alpha _{3} \cos \left( \omega +\pi \right) -1}=\frac{-\cos \omega -\alpha _{3}}{-\alpha _{3}\cos \omega -1}\nonumber \\&=-\frac{\cos \omega +\alpha _{3}}{-\alpha _{3}\cos \omega -1}=-\frac{c_{1}}{c_{2}}\left( \omega ,-\alpha _{3}\right) . \end{aligned}$$
  $$\square $$

Note that Lemma 2.8(1) implies that the curves $C_{0}$, $C_{k}^{\pm }$ can also be parameterized by $\alpha _{1}$. If we do so for $C_{0}$, we can in Lemma 2.7 replace “left” by “below” and “right” by “above”, just like in Lemma 2.6.

### **Lemma 2.9**

1. There are no intersections of the curves $C_{0},\ C_{k}^{\pm }$ .
2. The curves $C_{k}^{-}$ are situated in the quarter plane
  $$\begin{aligned} \left\{ \left( \alpha _{1},\alpha _{2}\right) :\alpha _{2}> \left| \alpha _{1}\right| \right\} , \end{aligned}$$
  while the curves $C_{0}$ and $C_{k}^{+}$ are situated in the quarter plane
  $$\begin{aligned} \left\{ \left( \alpha _{1},\alpha _{2}\right) :\alpha _{2}<- \left| \alpha _{1}\right| \right\} , \end{aligned}$$
  except for the starting point of $C_{0}$ which lies on the boundary.
3. The curves $C_{0},\ C_{k}^{\pm }$ are ordered as shown in Fig. 1.

### *Proof*

1. Assume that for some $\omega _{1}$ and $\omega _{2}$ it holds that
  $$\begin{aligned} \left( c_{1}\left( \omega _{1},\alpha _{3}\right) ,c_{2} \left( \omega _{1},\alpha _{3}\right) \right) =\left( c_{1}\left( \omega _{2},\alpha _{3}\right) ,c_{2}\left( \omega _{2},\alpha _{3}\right) \right) \end{aligned}$$
  then necessarily
  $$\begin{aligned} \frac{c_{1}\left( \omega _{1},\alpha _{3}\right) }{c_{2}\left( \omega _{1}, \alpha _{3}\right) }=\frac{c_{1}\left( \omega _{2},\alpha _{3}\right) }{c_{2}\left( \omega _{2},\alpha _{3}\right) } \end{aligned}$$
  i.e.,
  $$\begin{aligned} \frac{\cos \omega _{1}-\alpha _{3}}{\alpha _{3}\cos \omega _{1}-1}= \frac{\cos \omega _{2}-\alpha _{3}}{\alpha _{3}\cos \omega _{2}-1}. \end{aligned}$$
  Multiplying this identity by $(\alpha _{3}\cos \omega _{1}-1)(\alpha _{3}\cos \omega _{2}- 1)$ we find that necessarily $\cos \omega _{1}=\cos \omega _{2}$. By substituting $\cos \omega _{1}=\cos \omega _{2}$ in the identity $c_{1}(\omega _{1},\alpha _{3})=c_{1}(\omega _{2},\alpha _{3})$ we deduce that in addition the identity
  $$\begin{aligned} \frac{\omega _{1}}{\sin \omega _{1}}=\frac{\omega _{2}}{\sin \omega _{2}} \end{aligned}$$
  has to hold. But $\cos \omega _{1}=\cos \omega _{2}$ also implies that $\sin \omega _{1}=\pm \sin \omega _{2}$, so we arrive at the conclusion that $\omega _{1}=\pm \omega _{2}$. Since we parameterize with $\omega \ge 0$, the only possibility is $\omega _{1}=\omega _{2}$.
2. From Lemma 2.8(4) we know that for $\omega \not =0$
  $$\begin{aligned} -1<\frac{c_{1}}{c_{2}}<1. \end{aligned}$$
  If $c_{2}>0$ this amounts to $-c_{2}<c_{1}<c_{2}$, i.e., $c_{2}>c_{1}$ and $c_{2}>-c_{1}$. If $c_{2}<0$ it amounts to $-c_{2}>c_{1}>c_{2}$, i.e., $c_{2}<c_{1}$ and $c_{2}<-c_{1}$. In case of $C_{0}$ the inequality $-1<\frac{c_{1}}{c_{2}}$ holds for $\omega >0$ but not for $\omega =0$, where the inequality becomes equality.
3. Intersections of the curves with the $\alpha _{2}$-axis are characterized by $\cos \omega =\alpha _{3},$ cf (2.4a). So the corresponding value of $\alpha _{2}$ equals
  $$\begin{aligned} c_{2}\left( \omega ,\alpha _{3}\right) =\frac{\omega }{\sin \omega }\left( \alpha _{3}^{2}-1\right) \end{aligned}$$
  with $\sin \omega =\sqrt{1-\alpha _{3}^{2}}$ for $C_{0}$ and $C_{k}^{+}$ and $\sin \omega =-\sqrt{1-\alpha _{3}^{2}}$ for $C_{k}^{-}$. Accordingly the ordering is determined by $\omega ,$ so by *k*. $\square$

At last we are ready to present the

### *Proof of Theorem 2.1*

From Lemma 2.5(2) we know that at least a part of the negative $\alpha _{1}$-axis belongs to $S(\alpha _{3})$. As noted just before Lemma 2.4, the a priori estimate of Lemma 2.2 provides the compactness needed to apply Rouché’s theorem in order to deduce that $\partial S(\alpha _{3})$ is to a large extent characterized by (1.3) having a root on the imaginary axis. In other words, $\partial S(\alpha _{3})$ is contained in the union of the line $\alpha _{2}=-\alpha _{1}$ and the curves $C_{0}$, $C_{k}^{\pm }$, cf. Lemmas 2.4(1) and 2.4(2) and the definitions (2.3) and (2.11). On account of Lemma 2.9 we can now conclude that the connected component of $S(\alpha _{3})$ containing the negative $\alpha _{1}$-axis has $L(\alpha _{3})\cup C_{0}(\alpha _{3})$ as its boundary.

In principle $S(\alpha _{3})$ might have other components. Indeed, roots that enter the right half plane, when crossing $L(\alpha _{3})$ or $C_{0}(\alpha _{3})$, could return to the left half plane as one of the curves $C_{k}^{\pm }$ is crossed. There are at least two ways to see that, actually, this does not happen. The first is by extending Lemma 2.7 to the curves $C_{k}^{\pm }$; more precisely, by noting that the sign of $\text {det}M$ is opposite to the sign of $\sin \omega$ and that accordingly more and more roots move into the right half plane if we keep decreasing $\alpha _{2}$ after crossing $C_{0}$ or if we keep increasing $\alpha _{2}$ after crossing *L*. So also the numbers shown in Fig. 1 extend to $-1<\alpha _{3}<1$.

The second way is by showing that the roots that enter the right half plane upon crossing $C_{0}(\alpha _{3})$ remain “caught” in the strip $\{ \lambda :|\text {Im}\lambda |<\pi \}$, so cannot possibly return to the left half plane when one of the curves $C_{k}^{\pm }(\alpha _{3})$ is crossed. Indeed, let $\lambda =\mu +i\omega$ be a root of (1.3) and assume that $\mu \ge 0$ and $\sin \omega =0$. Then necessarily $\cos \omega =\pm 1$ and hence the equation $G_{2}=0$ (recall (2.9)) reads

$$\begin{aligned} -\omega \pm \alpha _{3}\omega e^{-\mu }=0 \end{aligned}$$

showing that either $\omega =0$ or $e^{-\mu }=\pm \frac{1}{\alpha _{3}}$. But since $\mu \ge 0$ we have $e^{-\mu }\le 1$ and since $-1<\alpha _{3}<1$ we have $|\pm \frac{1}{\alpha _{3}}|>1$, so $e^{-\mu }=\pm \frac{1}{\alpha _{3}}$ is not possible. (What can, and does happen though, is that they become real and that subsequently one of the two real roots returns to the left half plane when the parameter point crosses the line $\alpha _{2}=-\alpha _{1}$ from below to above at a point with $\alpha _{1}>1-\alpha _{3}$, cf. Lemma 2.6.)

We conclude that (1.3) has a root $\lambda$ with $\text {Re}\lambda >0$ if $(\alpha _{1},\alpha _{2})$ is in the connected open subset of ${\mathbb {R}}^{2}$ that has $L(\alpha _{3})\cup C_{0}(\alpha _{3})$ as its boundary and that does not contain the negative $\alpha _{1}$-axis. $\square$

It follows from Theorem 2.1 and Lemma 2.9(2) that the set

2.12 $$\begin{aligned} S_{u}:=\left\{ \left( \alpha _{1},\alpha _{2}\right) : \alpha _{1}\le \alpha _{2}<-\alpha _{1},\ \alpha _{1}<0\right\} \end{aligned}$$

belongs to $S(\alpha _{3})$ for $-1<\alpha _{3}<1$ (the index u is meant to express “uniformly” in $\alpha _{3}$). By analyzing the limiting behavior of $S(\alpha _{3})$ for $\alpha _{3}\uparrow 1$ and $\alpha _{3}\downarrow -1$, we shall show that this is sharp, i.e., $S_{u}$ is the largest set that belongs to $S(\alpha _{3})$ for $-1<\alpha _{3}<1$. Again we need some auxiliary results.

### **Lemma 2.10**

1. For $-\frac{1}{2}\le \alpha _{3}<1$ the inequality
  $$\begin{aligned} \frac{\partial c_{2}}{\partial \omega }\left( \omega ,\alpha _{3}\right) <0 \end{aligned}$$
  holds for $$0<\omega <\pi $$.
2. For $-1<\alpha _{3}<-\frac{1}{2}$ there exists $\theta (\alpha _{3})\in (0,\pi )$ such that
  $$\begin{aligned} \frac{\partial c_{2}}{\partial \omega }\left( \omega ,\alpha _{3}\right)&>0\quad \text {for }0<\omega <\theta \left( \alpha _{3}\right) \\ \frac{\partial c_{2}}{\partial \omega }\left( \omega ,\alpha _{3}\right)&<0\quad \text {for }\theta \left( \alpha _{3}\right) <\omega <\pi . \end{aligned}$$
3. $$\theta (\alpha _{3})\uparrow \pi $$ for $$\alpha _{3}\downarrow -1$$.

### *Proof*

1. $$\begin{aligned} \frac{\partial c_{2}}{\partial \omega }\left( \omega ,\alpha _{3}\right) = \frac{h(\omega ,\alpha _{3})}{\sin ^{2}\omega } \end{aligned}$$
  with
  2.13 $$\begin{aligned} h(\omega ,\alpha _{3}):=\alpha _{3}\left( \sin \omega \cos \omega - \omega \right) +\omega \cos \omega -\sin \omega . \end{aligned}$$
  Now note that $h(0,\alpha _{3})=0$ and that
  $$\begin{aligned} \frac{\partial h}{\partial \omega }(\omega ,\alpha _{3})=- \sin \omega \left( 2\alpha _{3}\sin \omega +\omega \right) <0 \quad \text {for }0<\omega <\pi \end{aligned}$$
  when $$\alpha _{3}\ge -\frac{1}{2}$$.
2. If $\alpha _{3}<-\frac{1}{2}$ we find from the above expression for $\frac{\partial h}{\partial \omega }$ that *h* increases for small positive $\omega$ and hence takes positive values for small positive $\omega$. Since $h(\pi ,\alpha _{3})=-(1+\alpha _{3})\pi <0$, *h* changes sign at least once on $(0,\pi )$ and the exact number of sign changes of *h* must be an odd number. If *h* changes sign three or more times, the derivative $\frac{\partial h}{\partial \omega }$ must also change sign at least three times. Now $\frac{\partial h}{\partial \omega }(\omega ,\alpha _{3})=0$ requires that $2\alpha _{3}\sin \omega +\omega =0$. We claim that whenever $2\alpha _{3}\sin \omega +\omega =0$ then necessarily $\frac{d}{d\omega }[2\alpha _{3}\sin \omega +\omega ]>0$. It follows that $2\alpha _{3}\sin \omega +\omega$ can change sign only once on $(0,\pi )$, that the same holds for $\frac{\partial h}{\partial \omega }$ and, therefore, for *h*. So *h* changes sign exactly once and since $\frac{\partial c_{2}}{\partial \omega }(\omega ,\alpha _{3})=\frac{h(\omega ,\alpha _{3})}{\sin ^{2}\omega }$ so does $\frac{\partial c_{2}}{\partial \omega }$. It remains to prove the claim. First observe that $\omega \cos \omega -\sin \omega <0$ since the left hand side equals zero for $\omega =0$ and $\frac{d}{d\omega }(\omega \cos \omega -\sin \omega )=- \omega \sin \omega <0$. Next note that
  $$\begin{aligned} \frac{d}{d\omega }\left( 2\alpha _{3}\sin \omega +\omega \right) = 2\alpha _{3}\cos \omega +1=\frac{2\alpha _{3}\sin \omega \cos \omega + \sin \omega }{\sin \omega }. \end{aligned}$$
  So if $2\alpha _{3}\sin \omega +\omega =0$ then
  $$\begin{aligned} \frac{d}{d\omega }\left( 2\alpha _{3}\sin \omega +\omega \right) = \frac{-\omega \cos \omega +\sin \omega }{\sin \omega }>0. \end{aligned}$$
3. $$\begin{aligned} h\left( \omega ,-1\right)&=-\sin \omega \cos \omega +\omega +\omega \cos \omega -\sin \omega \nonumber \\&=\left( 1+\cos \omega \right) \left( \omega -\sin \omega \right) \end{aligned}$$
  and both factors are positive on $(0,\pi )$. $\square$

For $\alpha _{3}\uparrow 1$ the point $(1-\alpha _{3},\alpha _{3}-1)$, where $L(\alpha _{3})$ and $C_{0}(\alpha _{3})$ meet, moves to (0, 0) and any point $(c_{1}(\omega ,\alpha _{3}),c_{2} (\omega ,\alpha _{3}))$ on $C_{0}(\alpha _{3})$ moves to $\frac{\omega (\cos \omega -1)}{\sin \omega }(1,1)$ on the line $\alpha _{1}=\alpha _{2}$. The fact that for $\frac{c_{1}}{c_{2}}$ one cannot interchange the limits $\omega \downarrow 0$ and $\alpha _{3}\uparrow 1$ is irrelevant, since the lines $\alpha _{1}=\alpha _{2}$ and $\alpha _{1}=-\alpha _{2}$ intersect in (0, 0). Note that $\frac{\omega (\cos \omega -1)}{\sin \omega }$ decreases strictly from 0 to $-\infty$ as $\omega$ increases from 0 to $\pi$, see Lemma 2.8(1).

For $\alpha _{3}\downarrow -1$, the point $(1-\alpha _{3},\alpha _{3}-1)$ moves to $(2,-2)$ and any point $(c_{1}(\omega ,\alpha _{3})$, $c_{2}(\omega ,\alpha _{3}))$ on $C_{0}(\alpha _{3})$ moves to $\frac{\omega (\cos \omega +1)}{\sin \omega } (1,-1)$ on the line $\alpha _{1}=-\alpha _{2}$. In this case, one cannot interchange the limits $\omega \uparrow \pi$ and $\alpha _{3}\downarrow -1$. For $\alpha _{3}$ slightly above $-1$, there is a small interval $(\theta (\alpha _{3}),\pi )$ such that $(c_{1}(\omega ,\alpha _{3}),c_{2}(\omega ,\alpha _{3}))$ moves from close to (0, 0) for $\omega =\theta (\alpha _{3})$ all the way to $-\infty (1,1)$ for $\omega =\pi$, see Lemma 2.10. So the limit set of $C_{0}(\alpha _{3})$ is the union of $\{ (\gamma ,\gamma ):-\infty <\gamma \le 0\}$ and $\{ (\gamma ,-\gamma ):0\le \gamma \le 2\}$. Or, in other words, $S(\alpha _{3})$ converges to $S_{u}$, but if we use $\omega$ to parameterize the $C_{0}(\alpha _{3})$ part of the boundary, the convergence is rather non-uniform. We summarize our conclusions in

### **Theorem 2.11**

1. $$S_{u}=\cap _{-1<\alpha _{3}<1}S(\alpha _{3})$$
2. If $(\alpha _{1},\alpha _{2})\in S(\alpha _{3})$ for all $\alpha _{3}$ in an interval of either the form $(-1,-1+\varepsilon )$ or of the form $(1-\varepsilon ,1)$ for some $\varepsilon >0$, then $(\alpha _{1},\alpha _{2})\in S_{u}$.

For completeness, we formulate a result about the limiting behaviour of the curves $C_{k}^{\pm }$. As a first indication of what to expect, recall from the proof of Lemma 2.9(3) that the intersection with the $\alpha _{2}$-axis has

$$\begin{aligned} c_{2}\left( \omega ,\alpha _{3}\right) =\frac{\omega }{\sin \omega } \left( \alpha _{3}^{2}-1\right) \end{aligned}$$

and conclude that the intersection point converges to (0, 0). As in the proof of Lemma (2.10)(2) one can show that $\frac{\partial c_{2}}{\partial \omega }$ changes sign exactly once on $I_{k}^{\pm }$ defined in (2.10). So geometrically it seems clear that the bounds from Lemma 2.9(2) become sharp in the limit as $|\alpha _{3}|$ converges to 1. We now prove that this is indeed the case.

### **Lemma 2.12**

When either $\alpha _{3}\uparrow 1$ or $\alpha _{3}\downarrow -1$, the curves $C_{k}^{-}$ all converge to

$$\begin{aligned} \left\{ \left( \alpha _{1},\alpha _{2}\right) :\alpha _{2}= \left| \alpha _{1}\right| ,\ -\infty <\alpha _{1}<\infty \right\} \end{aligned}$$

while the curves $C_{k}^{+}$ all converge to

$$\begin{aligned} \left\{ \left( \alpha _{1},\alpha _{2}\right) :\alpha _{2}=-\left| \alpha _{1} \right| ,\ -\infty <\alpha _{1}<\infty \right\} . \end{aligned}$$

### *Proof*

We provide the proof for $C_{1}^{-}$ and $\alpha _{3}\uparrow 1$, in order to illustrate some details more clearly. We trust that the reader believes that all the other cases are covered by essentially the same arguments.

The curve $C_{1}^{-}$ is parameterized by $\omega \in I_{1}^{-}= (\pi ,2\pi )$. So $\sin \omega <0$ and $\cos \omega$ increases from $-1$ to $+1$. From (2.4) we conclude that for any fixed $\omega$ both $c_{1}$ and $c_{2}$ converge to $\frac{\omega }{\sin \omega }(\cos \omega -1)>0$ as $\alpha _{3}\uparrow 1$. So $(c_{1},c_{2})$ converges to a point on the half-line $\alpha _{2}=\alpha _{1}$, $\alpha _{1}>0$. Since $\frac{\omega }{\sin \omega }(\cos \omega -1)$ decreases from $+\infty$ for $\omega \downarrow \pi$ to 0 for $\omega \uparrow 2\pi$, any point on this line is in fact the limit of points on $C_{1}^{-}(\alpha _{3})$ as $\alpha _{3}\uparrow 1$.

Let *r* be an arbitrary negative real number. By Lemma 2.8(2) we know that $\omega =\omega (\alpha _{3},r)$ exists such that $c_{1}(\omega ,\alpha _{3})=r$. Note that we must have $\cos \omega -\alpha _{3}>0$ for $\omega =\omega (\alpha _{3},r)$. Hence $\omega (\alpha _{3},r)\uparrow 2\pi$ as $\alpha _{3}\uparrow 1$. So let us put $\omega (\alpha _{3},r)=2\pi -\varepsilon$ where, of course, $\varepsilon =\varepsilon (\alpha _{3},r)$. Using Taylor expansion of $\sin \omega$ and $\cos \omega$, we find from the equation $c_{1}(\omega ,\alpha _{3})=r$ that $\varepsilon =\frac{2\pi (1-\alpha _{3})}{-r}+o(1-\alpha _{3})$. Next the expression for $c_{2}$ yields $c_{2}=\frac{2\pi +O(\varepsilon )}{-\varepsilon +O (\varepsilon ^{2})} (\alpha _{3}-1-\frac{1}{2}\alpha _{3}\varepsilon ^{2}+O(\varepsilon ^{4}))=-r+o(1-\alpha _{3})$. We conclude that $(c_{1}(\omega (\alpha _{3},r),\alpha _{3}), c_{2}(\omega (\alpha _{3},r),\alpha _{3}))$ converges to $(r,-r)$ as $\alpha _{3}\uparrow 1$. $\square$

**Fig. 3:**
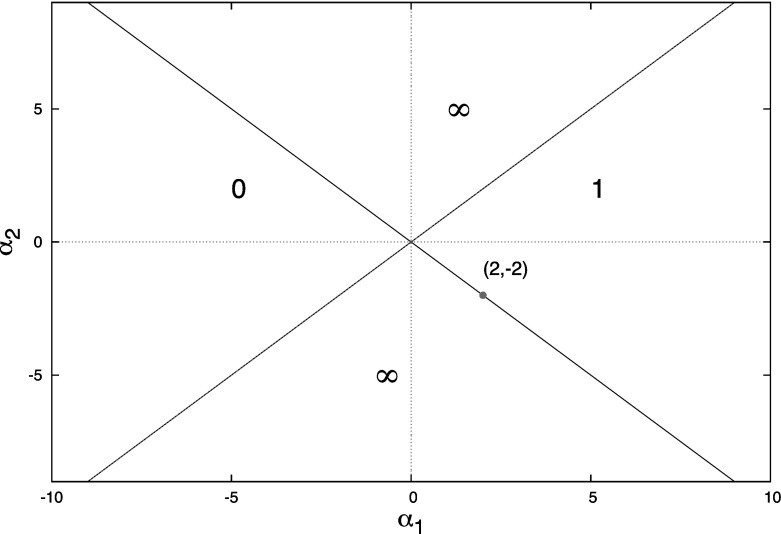
Partitioning of the $(\alpha _{1},\alpha _{2})$-parameter plane according to the number of roots of (1.3) in the right half plane for the limiting cases $\alpha _{3}=+1$ and $\alpha _{3}=-1$

The limiting stability diagram is depicted in Fig. 3. Lemma 2.12 and this diagram are in complete accordance with the results that one obtains by studying (1.3) directly for $\alpha _{3}=\pm 1$. The two real equations

$$\begin{aligned} \omega&=-\alpha _{2}\sin \omega \pm \omega \cos \omega \\ 0&=\alpha _{1}+\alpha _{2}\cos \omega \pm \omega \sin \omega \end{aligned}$$

yield that for $\omega \not =k\pi$

$$\begin{aligned} \alpha _{2}&=\frac{\omega }{\sin \omega }\left( \pm \cos \omega -1\right) \\ \alpha _{1}&=\pm \alpha _{2} \end{aligned}$$

while putting $\omega =k\pi$ yields

$$\begin{aligned} k\pi&=\pm k\pi \left( -1\right) ^{k}\\ 0&=\alpha _{1}\pm \alpha _{2}\left( -1\right) ^{k}. \end{aligned}$$

So $\alpha _{3}=+1$ requires *k* to be even and then $0=\alpha _{1}+\alpha _{2}$ while $\alpha _{3}=-1$ requires *k* to be odd and then $0=\alpha _{1}-\alpha _{2}$.

## A cell population model involving quiescence

Motivated by Alarcón et al. (2014), we assume that the cell cycle incorporates a checkpoint for the prevailing environmental condition $E=E(t)$ in the sense that, upon passage of this point, a cell commits itself to division with probability $\beta _{1}=\beta _{1}(E)$, while going quiescent with probability $\beta _{2}=\beta _{2}(E)$. If we allow that $\beta _{1}+\beta _{2}<1$ one can interpret $1-\beta _{1}-\beta _{2}$ as the probability that the cell undergoes checkpoint triggered apoptosis. Here, however, we shall assume that $\beta _{1}+\beta _{2}=1$ and that $\beta _{1}\in [0,1)$.

Quiescent cells have a probability per unit of time $G=G(E)$ to reactivate and then progress towards division. Once reactivated, they differ in no way from the cells that never went quiescent. So quiescence amounts to a variable delay determined by the course of the environmental condition and an element of chance.

We assume that the proliferation step takes a fixed amount of time $\tau$, in the following sense:

1. if a cell commits itself to proliferation at the checkpoint, its (surviving) offspring arrives at the checkpoint after time $\tau$
2. if a quiescent cell is reactivated, it likewise takes exactly time $\tau$ for its offspring to arrive at the checkpoint.

A dividing cell produces two daughter cells. But we allow for a uniform death rate $\mu \ge 0$ and accordingly the expected number of progeny arriving at the checkpoint after time $\tau$ equals $2\exp (-\mu \tau )$. See Alarcón et al. (2014) for a variant that incorporates a more general probability distribution for cell cycle duration as well as a more general survival probability. Also see Adimy and Chekroun (Adimy and Chekroun) for a detailed analysis of the situation that $\beta _{1}$ does not depend on *E* and for references to various papers on hematopoietic cell models, many of them originating from the pioneering work of Mackey (1978).

Let *Q*(*t*) denote the quantity of quiescent cells at time *t*. Let *p*(*t*) denote the quantity of cells that, per unit of time, set out on division at time *t*. The mathematical formulation of the assumptions described above takes the form

3.1a $$\begin{aligned} p(t)&=2\beta _{1}\left( E(t)\right) e^{-\mu \tau }p(t-\tau )+ G(E(t))Q(t), \end{aligned}$$

3.1b $$\begin{aligned} \frac{dQ(t)}{dt}&=2\beta _{2}\left( E(t)\right) e^{-\mu \tau }p(t-\tau )-\left( \mu +G(E(t))\right) Q(t). \end{aligned}$$

To complete the model formulation, we need to specify the dynamics of *E* and, in particular, the feedback law that describes the impact of the cell population on the environmental condition.

For concreteness, think of *E* as oxygen concentration. The equation

3.2 $$\begin{aligned} \frac{dE(t)}{dt}=c_{\mathrm{in}}E_{\mathrm{in}}-\left( c_{\mathrm{out}}+ c_{p}\int _{0}^{\tau }p(t-a)e^{-\mu a}da+c_{q}Q(t)\right) E(t) \end{aligned}$$

expresses that *E* is determined by the balance of inflow, outflow and consumption. If the constants $c_{\mathrm{in}},\ c_{\mathrm{out}},\ c_{p}$ and $c_{q}$ are all big relative to $\mu$ and the range of $\beta _{1},\ \beta _{2}$ and *G*, we can make a quasi-steady-state-assumption and replace (3.2) by the explicit expression for *E*, in terms of *Q* and the history of *p*, obtained by putting the right hand side of (3.2) equal to zero and solving for *E*. Before writing this expression, let us discuss the issue of scaling and the concomitant reduction in the number of parameters.

By scaling of time, we can achieve that $\tau =1$. Since the system (3.1) is linear in *p* and *Q*, it does not change when we scale both of these variables with the same factor. A particular choice of this factor amounts to replacing $c_{p}$ by $\theta c_{\mathrm{out}}$ and $c_{q}$ by $(1-\theta )c_{\mathrm{out}}$ with $0\le \theta \le 1$. Finally, in (3.1) the variable *E* only occurs as an argument of $\beta _{1},\ \beta _{2}$ and *G* and we have not yet specified these functions. So scaling *E* by the factor $\frac{c_{\mathrm{in}}}{c_{\mathrm{out}}}E_{\mathrm{in}}$ does not lead to any loss of generality. The upshot is the scaled system

3.3a $$\begin{aligned} p(t)&=2\beta _{1}\left( E(t)\right) e^{-\mu }p(t-1)+G(E(t))Q(t), \end{aligned}$$

3.3b $$\begin{aligned} \frac{dQ(t)}{dt}&=2\beta _{2}\left( E(t)\right) e^{-\mu }p(t-1)-\left( \mu +G(E(t))\right) Q(t), \end{aligned}$$

3.3c $$\begin{aligned} E(t)&=\frac{1}{1+\theta \int _{0}^{1}p(t-a)e^{-\mu a}da+\left( 1-\theta \right) Q(t)}, \end{aligned}$$

that is based on the quasi-steady-state-assumption. The remaining parameters are $\mu ,\ \theta$ and the two functions *G* and $\beta _{1}$ (note carefully that throughout the rest of the paper $\beta _{2}=1-\beta _{1}$).

Our main general results concerning (3.3) are

Theorem 3.1 below about existence and uniqueness of a nontrivial steady state
(in)stability of the nontrivial steady state is determined by the position in ${\mathbb {C}}$ of the roots of (1.3) with $\alpha$ given by (3.20) below

We use the systematic methodology of Diekmann et al. (2003) to derive Theorem 3.1. Linearization in the trivial steady state amounts to putting $E(t)\equiv 1$ in the equations for *p* and *Q*. So the linearized system reads (when, abusing notation, we use the same symbols to denote the variables)

3.4a $$\begin{aligned} p(t)&=2\beta _{1}\left( 1\right) e^{-\mu }p(t-1)+ G(1)Q(t), \end{aligned}$$

3.4b $$\begin{aligned} \frac{dQ(t)}{dt}&=2\beta _{2}\left( 1\right) e^{-\mu }p(t-1) -\left( \mu +G(1)\right) Q(t). \end{aligned}$$

In order to reveal the link between the (in)stability of the trivial steady state and the (non)existence of a nontrivial steady state, consider, for fixed positive *E*, the linear system

3.5a $$\begin{aligned} p(t)&=2\beta _{1}\left( E\right) e^{-\mu }p(t-1)+G(E)Q(t), \end{aligned}$$

3.5b $$\begin{aligned} \frac{dQ(t)}{dt}&=2\beta _{2}\left( E\right) e^{-\mu }p(t-1)- \left( \mu +G(E)\right) Q(t). \end{aligned}$$

At steady state the value *E* should be such that (3.5) has a nontrivial (and positive) steady state. If it has, it has a one-parameter family (since (3.5) is a linear system). The parameter should then be tuned so that *E*, when expressed by the steady state version of (3.3c), does have the required value.

So we study the linear system (3.5) with parameter *E*. The simplest approach is based on the biological interpretation and runs as follows. In a constant environment a cell that goes quiescent has probability

$$\begin{aligned} \frac{G(E)}{\mu +G(E)} \end{aligned}$$

to become reactivated (rather than dying while quiescent). Hence the overall probability that a cell arriving at the checkpoint sets out on division, equals

$$\begin{aligned} \beta _{1}(E)+\beta _{2}(E)\frac{G(E)}{\mu +G(E)}= \frac{\beta _{1}(E)\mu +G(E)}{\mu +G(E)}. \end{aligned}$$

Accordingly the expected number $R_{0}(E)$ of progeny arriving at the checkpoint is given by the formula

3.6 $$\begin{aligned} R_{0}(E)=2e^{-\mu }\frac{\beta _{1}(E)\mu +G(E)}{\mu +G(E)}. \end{aligned}$$

On the basis of (3.4) we now conclude that the trivial steady state is stable if $R_{0}(1)<1$ and unstable if $R_{0}(1)>1$.

If either

$$\begin{aligned} \beta _{1}\text { is strictly increasing and }G\text { is non-decreasing} \end{aligned}$$

or

$$\begin{aligned} \beta _{1}\text { is non-decreasing and }G\text { is strictly increasing} \end{aligned}$$

we find (by calculating the derivative of the right hand side of (3.6)) that $R_{0}$ is a strictly increasing function of *E*.

In that case, there is at most one root for the equation

3.7 $$\begin{aligned} R_{0}(E)=1. \end{aligned}$$

The Eq. (3.3c) shows that only roots in [0, 1] yield candidates for steady states. The assumption

3.8 $$\begin{aligned} R_{0}\left( 1\right) >1 \end{aligned}$$

guarantees that the unique root, if it exists, belongs to [0, 1). The existence is indeed guaranteed if, in addition to (3.8), we assume that

3.9 $$\begin{aligned} R_{0}\left( 0\right) <1. \end{aligned}$$

Incidentally, note that $R_{0}(1)>1$ cannot possibly hold if $2e^{-\mu }\le 1$, so by imposing (3.8) we implicitly require that $2e^{-\mu }>1$. Also note that from (3.6) it follows that $R_{0}(E)>2e^{-\mu }\beta _{1}(E)$ and hence we automatically have

3.10 $$\begin{aligned} 2e^{-\mu }\beta _{1}\left( E\right) <1 \end{aligned}$$

if $$R_{0}(E)=1$$.

We denote by ${\overline{E}}$ the unique solution of (3.7). Let us look for constant solutions of (3.5). These should satisfy

$$\begin{aligned} \left( \begin{array}{c@{\quad }c} 1-2e^{-\mu }\beta _{1}\left( {\overline{E}}\right) &{} -G({\overline{E}})\\ 2e^{-\mu }\beta _{2}\left( {\overline{E}}\right) &{} -\left( \mu +G({\overline{E}})\right) \end{array}\right) \left( \begin{array}{c} p\\ Q \end{array}\right) =\left( \begin{array}{c} 0\\ 0 \end{array}\right) . \end{aligned}$$

The determinant of the matrix is equal to

$$\begin{aligned} \left( \mu +G({\overline{E}})\right) \left( R_{0}\left( {\overline{E}}\right) -1\right) \end{aligned}$$

so we see right away that the condition $R_{0}(E)=1$ for $E={\overline{E}}$ is both necessary and sufficient for a solution to exist. Then the vector

3.11 $$\begin{aligned} \left( \begin{array}{c} {\overline{p}}\\ {\overline{Q}} \end{array}\right) =K\left( \begin{array}{c} G({\overline{E}})\\ 1-2e^{-\mu }\beta _{1}({\overline{E}}) \end{array}\right) \end{aligned}$$

is, for any real number *K*, in the null space of the matrix, so is a constant solution of (3.5). Finally, we close the feedback loop by requiring that

$$\begin{aligned} {\overline{E}}=\frac{1}{1+K\left\{ \theta G({\overline{E}}) \frac{1-e^{-\mu }}{\mu }+\left( 1-\theta \right) \left( 1-2e^{-\mu }\beta _{1} ({\overline{E}})\right) \right\} }. \end{aligned}$$

Since $$R_{0}({\overline{E}})=1$$ iff

3.12 $$\begin{aligned} G({\overline{E}})=\frac{1-2\beta _{1}({\overline{E}}) e^{-\mu }}{2e^{-\mu }-1}\mu \end{aligned}$$

holds, one has

$$\begin{aligned} {\overline{E}}=\frac{1}{1+K\left( 1-2e^{-\mu } \beta _{1}({\overline{E}})\right) \left( 1-l\theta \right) }, \end{aligned}$$

where we define

3.13 $$\begin{aligned} l:=\frac{3e^{-\mu }-2}{2e^{-\mu }-1}\in \left( -\infty ,1\right) . \end{aligned}$$

We then find that

3.14 $$\begin{aligned} K=\left( \frac{1}{{\overline{E}}}-1\right) \frac{1}{\left( 1-2e^{-\mu }\beta _{1}\left( {\overline{E}}\right) \right) \left( 1-l\theta \right) }. \end{aligned}$$

Using (3.11)–(3.14) one obtains ${\overline{p}}$ and ${\overline{Q}}$ as

3.15 $$\begin{aligned} \left( \begin{array}{c} {\overline{p}}\\ {\overline{Q}} \end{array}\right) =\left( \frac{1}{{\overline{E}}}-1\right) \frac{1}{1-l\theta }\left( \begin{array}{c} \frac{\mu }{2e^{-\mu }-1}\\ 1 \end{array}\right) . \end{aligned}$$

We summarize the discussion above in

### **Theorem 3.1**

Let $\beta _{1}$ and *G* be continuously differentiable functions such that, for $0<E<1$,

$$\begin{aligned} \beta _{1}^{\prime }(E)(\mu +G(E))+G^{\prime }(E)(1-\beta _{1}(E))>0. \end{aligned}$$

Assume that, with $R_{0}(E)$ defined by (3.6),

3.16 $$\begin{aligned} R_{0}(0)<1<R_{0}(1). \end{aligned}$$

Then Eq. (3.7) has a unique solution $E={\overline{E}}$. Moreover, (3.3) has a unique steady state with *p* and *Q* given by (3.15).

### *Remark 3.2*

It is biologically obvious that if $R_{0}(1)<1$ the population goes extinct, while if $R_{0}(0)>1$ it grows beyond any bound, despite the negative feedback via $\beta _{1}$ and/or *G*. A proof is provided in the Appendix.

Our next aim is to study the stability of the nontrivial steady state. The first step in this direction consists of linearization of system (3.3). We put

$$\begin{aligned} p(t)&={\overline{p}}+x(t)\\ Q(t)&={\overline{Q}}+y(t) \end{aligned}$$

and focus on small*x*, *y*. The Eq. (3.3c) implies that

$$\begin{aligned} E(t)&=\frac{1}{\left( {\overline{E}}\right) ^{-1}+\theta \int _{0}^{1} x(t-a)e^{-\mu a}da+\left( 1-\theta \right) y(t)}\nonumber \\&={\overline{E}}-{\overline{E}}^{2}\left\{ \theta \int _{0}^{1} x(t-a)e^{-\mu a}da+\left( 1-\theta \right) y(t)\right\} +\text {h.o.t.} \end{aligned}$$

For $f=\beta _{1},\beta _{2},G$ we therefore have

$$\begin{aligned} f(E(t))=f({\overline{E}})-f^{\prime }({\overline{E}}) {\overline{E}}^{2}\left\{ \theta \int _{0}^{1}x(t-a)e^{-\mu a}da+\left( 1-\theta \right) y(t)\right\} +\text {h.o.t.} \end{aligned}$$

Thus we deduce that the linearized system is given by

$$\begin{aligned} x(t)&= 2e^{-\mu }\beta _{1}\left( {\overline{E}}\right) x(t-1)\nonumber \\&\quad -\left( 2e^{-\mu }\beta _{1}^{\prime }({\overline{E}}){\overline{p}}+G^{\prime }({\overline{E}}){\overline{Q}}\right) {\overline{E}}^{2}\theta \int _{0}^{1}x(t-a)e^{-\mu a}da\nonumber \\&\quad +\left\{ G({\overline{E}})-\left( 2e^{-\mu }\beta _{1}^{\prime }\left( {\overline{E}}\right) {\overline{p}}+G^{\prime }\left( {\overline{E}}\right) {\overline{Q}}\right) {\overline{E}}^{2}\left( 1-\theta \right) \right\} y(t),\\ \frac{dy}{dt}(t)&=2e^{-\mu }\beta _{2}\left( {\overline{E}} \right) x(t-1)\nonumber \\&\quad -\left( 2e^{-\mu }\beta _{2}^{\prime }({\overline{E}}){\overline{p}}- G^{\prime }({\overline{E}}){\overline{Q}}\right) {\overline{E}}^{2} \theta \int _{0}^{1}x(t-a)e^{-\mu a}da\nonumber \\&\quad -\left\{ \mu +G({\overline{E}})+\left( 2e^{-\mu }\beta _{2}^{\prime } \left( {\overline{E}}\right) {\overline{p}}-G^{\prime } \left( {\overline{E}}\right) {\overline{Q}}\right) {\overline{E}}^{2} \left( 1-\theta \right) \right\} y(t). \end{aligned}$$

Define

3.17 $$\begin{aligned} A({\overline{E}}){:=}\left( \frac{2e^{-\mu }\mu }{2e^{-\mu }-1} \beta _{1}^{\prime }({\overline{E}})+G^{\prime }({\overline{E}})\right) {\overline{E}}\left( 1-{\overline{E}}\right) . \end{aligned}$$

Then from (3.15)

$$\begin{aligned} \left( 2e^{-\mu }\beta _{1}^{\prime }({\overline{E}}){\overline{p}}+ G^{\prime }({\overline{E}}){\overline{Q}}\right) {\overline{E}}^{2}= A({\overline{E}})\frac{1}{1-l\theta }. \end{aligned}$$

Recall that $\beta _{1}+\beta _{2}=1$ (and hence $\beta _{2}^{\prime }({\overline{E}})=- \beta _{1}^{\prime }({\overline{E}})$).

The characteristic equation has the form

3.18 $$\begin{aligned} \text {det}M(\lambda )=0 \end{aligned}$$

where $M(\lambda )$ is the $2\times 2$ matrix with entries

3.19a $$\begin{aligned} M_{11}(\lambda )&=1-2e^{-\mu }\beta _{1}\left( {\overline{E}} \right) e^{-\lambda }+A({\overline{E}})\frac{\theta }{1-l\theta } \frac{1-e^{-\left( \mu +\lambda \right) }}{\mu +\lambda }, \end{aligned}$$

3.19b $$\begin{aligned} M_{12}(\lambda )&=-G({\overline{E}})+A({\overline{E}}) \frac{1-\theta }{1-l\theta }, \end{aligned}$$

3.19c $$\begin{aligned} M_{21}(\lambda )&=2e^{-\mu }\left( \beta _{1}\left( {\overline{E}} \right) -1\right) e^{-\lambda }-A({\overline{E}})\frac{\theta }{1- l\theta }\frac{1-e^{-\left( \mu +\lambda \right) }}{\mu +\lambda }, \end{aligned}$$

3.19d $$\begin{aligned} M_{22}(\lambda )&=\lambda +\mu +G({\overline{E}})-A({\overline{E}}) \frac{1-\theta }{1-l\theta }. \end{aligned}$$

A straightforward calculation now establishes that (3.18) is exactly of the form (1.3) with

3.20a $$\begin{aligned} \alpha _{1}&=-\mu -G({\overline{E}})+A({\overline{E}}) \frac{1-2\theta }{1-l\theta } \end{aligned}$$

3.20b $$\begin{aligned} \alpha _{2}&=2e^{-\mu }\left( \mu \beta _{1}({\overline{E}}) +G({\overline{E}})+A({\overline{E}})\frac{\frac{3}{2}\theta -1}{1-l\theta }\right) , \end{aligned}$$

3.20c $$\begin{aligned} \alpha _{3}&=2e^{-\mu }\beta _{1}\left( {\overline{E}} \right) . \end{aligned}$$

Note that $0<\alpha _{3}<1$ (recall (3.10)). A straightforward computation, using (3.12) and (3.13), establishes that

3.21 $$\begin{aligned} \alpha _{1}+\alpha _{2}=\left( 1-2e^{-\mu }\right) A({\overline{E}}). \end{aligned}$$

As observed in between (3.9) and (3.10) the first factor is negative. The expression (3.17) shows that $A({\overline{E}})$ is positive if $0<{\overline{E}}<1$. We conclude that $\alpha _{1}+\alpha _{2}<0$ for $0<{\overline{E}}<1$ and that $\alpha _{1}+\alpha _{2}=0$ corresponds to the transcritical bifurcation at which $(p,Q)=(0,0)$ (and hence $E=1$) loses its stability (in principle $\alpha _{1}+\alpha _{2}=0$ could yield saddle-node bifurcations as well, but since the internal equilibrium is unique, these do not actually happen).

## The impact of $\theta$ (a one parameter study)

Proliferating and quiescent cells consume, in the model, oxygen in the proportion $\theta :1-\theta$. Here we allow in principle $0\le \theta \le 1$, but a more reasonable range is $\frac{1}{2}\le \theta \le 1$ (since if quiescent cells would consume more oxygen than proliferating cells, there does not seem to be any benefit in going quiescent). The aim of this section is to investigate the impact of $\theta$ on the stability of the nontrivial steady state.

As $R_{0}$ does not depend on $\theta$, neither does ${\overline{E}}$. The formulas (3.20c) and (3.21) above next show that both $\alpha _{3}$ and $\alpha _{1}+\alpha _{2}$ remain constant when $\theta$ is varied. Since $l<1$ (recall (3.13)), $\alpha _{1}$ decreases if $\theta$ is increased, so increasing $\theta$ amounts to moving North-West in the $(\alpha _{1},\alpha _{2})$-plane along the line defined by (3.21).

### **Proposition 4.1**

For $\theta \in [\frac{2}{3},1]$ the nontrivial steady state is asymptotically stable.

### *Proof*

By inserting $\theta =\frac{2}{3}$ in (3.20b) we find $\alpha _{2}>0$. Since $\alpha _{1}+\alpha _{2}<0$, the corresponding point is located in the 2nd quadrant of the $(\alpha _{1},\alpha _{2})$-plane, which belongs to $S(\alpha _{3})$. If we increase $\theta$ we move along the line defined by (3.21) away from its intersection with $C_{0}(\alpha _{3})$ defined by (2.3), so we remain in $S(\alpha _{3})$. $\square$

The intersection of the line (3.21) and the curve $C_{0}(\alpha _{3})$ is found by solving the equation

4.1 $$\begin{aligned} \frac{\omega }{\sin \omega }\left( \cos \omega -1\right) = \frac{1-2e^{-\mu }}{1+2e^{-\mu }\beta _{1}({\overline{E}})}A({\overline{E}}) \end{aligned}$$

for $\omega \in (0,\pi )$. Note that $\frac{\omega }{\sin \omega }(\cos \omega -1)$ decreases from 0 for $\omega =0$ to $-\infty$ for $\omega =\pi$. Hence, since $1-2e^{-\mu }<0$, a unique solution exists.

Once the root of (4.1) is found, we can compute the corresponding value of $\alpha _{1}$ by inserting the root into (2.4a). Let us call the result ${\overline{\alpha }}_{1}$. The value of $\alpha _{1}$ corresponding to $\theta =\frac{1}{2}$ is, according to (3.20a), given by $-\mu -G({\overline{E}})$. This provides us with a simple test:

1. if $-\mu -G({\overline{E}})<{\overline{\alpha }}_{1}$ the nontrivial steady state is asymptotically stable for $\theta \in [\frac{1}{2},1]$.
2. if $-\mu -G({\overline{E}})>{\overline{\alpha }}_{1}$ there exists $\theta _{\mathrm{crit}}\in (\frac{1}{2},\frac{2}{3})$ such that the nontrivial steady state is asymptotically stable for $\theta \in (\theta _{\mathrm{crit}},1]$ but unstable for $\theta \in [\frac{1}{2},\theta _{\mathrm{crit}})$.

In the next section we show that it is far more efficient (as well as far more illuminating) to implement a two parameter version of this test. For now we just conclude that increasing $\theta$ promotes stability of the nontrivial steady state.

## Two-parameter case studies

The model described in Sect. 3 involves two regulatory mechanisms: the partitioning between quiescent and proliferating cells at the checkpoint, as described by the dependence of $\beta _{1}$ on *E*, and the duration of the quiescent period, as described by the dependence of *G* on *E*. We will study these mechanisms separately and in turn, meaning that we first consider the case that $\beta _{1}$ does not depend on *E* and next the case that *G* does not depend on *E*. We use respectively $G^{\prime }({\overline{E}})$ and $\beta _{1}^{\prime }({\overline{E}})$ as a parameter, which measures how strongly the system reacts locally near the steady state to changes in the environmental condition.

The feedback to the environmental condition is described by Eq. (3.3c). The parameter $\theta$ captures the relative impact on the environmental condition of proliferating and quiescent cells. We will take $\theta$ as the second parameter.

As shown below, in both cases $\alpha _{3}$, as defined by (3.20c), does not depend on $\theta$. Our strategy will be to use (3.20a) and (3.20b), and the definition of *A* and ${\overline{E}}$, in order to express $G^{\prime }({\overline{E}})$ (or $\beta _{1}^{\prime }({\overline{E}})$) and $\theta$ in terms of $\alpha _{1}$ and $\alpha _{2}$. These expressions then allow us to picture the stability boundary in the $(G^{\prime }({\overline{E}}),\theta )$-plane by mapping $L(\alpha _{3})$ and $C_{0}(\alpha _{3})$ as defined in, respectively, (2.2) and (2.3), to the $(G^{\prime }({\overline{E}}),\theta )$-plane.

The following observations are inspired by the analysis of Sect. 4. The shape of $S(\alpha _{3})$ shows that instability is promoted by letting $\alpha _{1}$ increase and $\alpha _{2}$ decrease i.e., by moving South-East in the $(\alpha _{1},\alpha _{2})$-plane. If $\theta <\frac{2}{3}$ then $\alpha _{2}$ defined by (3.20b) decreases if $A({\overline{E}})$ increases. Such an increase of $A({\overline{E}})$ has, for $\theta >\frac{1}{2}$, the effect that $\alpha _{1}$ decreases as well. But for $\theta$ only slightly bigger than $\frac{1}{2}$ the effect on $\alpha _{2}$ is much larger than the effect on $\alpha _{1}$, so we might expect that making $A({\overline{E}})$ bigger leads to instability. Next (3.17) tells us that having either $\beta _{1}^{\prime }$ or $G^{\prime }$ large in $E={\overline{E}}$ is expected to promote instability. In a nutshell: we expect that steep response promotes instability.

### Regulation via the length of the quiescent period

We assume that $\beta _{1}$ is independent of *E*. It follows right away from (3.20c) that $\alpha _{3}$ is a constant depending on $\mu$ and $\beta _{1}$, but not on $\theta$. We fix $\beta _{1}$ and $\mu$ such that (3.10) holds and assume that the strictly increasing function *G* is such that

$$\begin{aligned} G(0)<\frac{\left( 1-2\beta _{1}e^{-\mu }\right) \mu }{2e^{-\mu }- 1}=\frac{\left( 1-\alpha _{3}\right) \mu }{2e^{-\mu }-1}<G(1), \end{aligned}$$

holds, which is equivalent to assuming (3.16). Using (3.6) and (3.20c) we write (3.7) in the form

5.1 $$\begin{aligned} G({\overline{E}})=\frac{\left( 1-\alpha _{3}\right) \mu }{ 2e^{-\mu }-1} \end{aligned}$$

and accordingly define

5.2 $$\begin{aligned} {\overline{E}}=G^{-1}\left( \frac{\left( 1-\alpha _{3}\right) \mu }{2e^{-\mu }-1}\right) . \end{aligned}$$

If we eliminate *A* from the pair of equations (3.20a) and (3.20b), we obtain the identity

$$\begin{aligned} \left( \frac{3}{2}\theta -1\right) \left( \alpha _{1}+ \mu +G({\overline{E}})\right) =\left( 1-2\theta \right) \left( \frac{\alpha _{2}-\mu \alpha _{3}}{2e^{-\mu }}-G({\overline{E}})\right) . \end{aligned}$$

Solving for $\theta$ we find

5.3 $$\begin{aligned} \theta =\frac{\alpha _{2}-\mu \alpha _{3}+2e^{-\mu }\left( \alpha _{1} +\mu \right) }{3e^{-\mu }\left( \alpha _{1}+\mu \right) +2\left( \alpha _{2}-\mu \alpha _{3}\right) -e^{-\mu }G({\overline{E}})} \end{aligned}$$

which upon substitution of (5.1) becomes an explicit expression for $\theta$ in terms of $\alpha _{1}$, $\alpha _{2}$, $\alpha _{3}$ and $\mu$.

When $\beta _{1}$ does not depend on *E*, (3.17) simplifies to

$$\begin{aligned} A({\overline{E}})=G^{\prime }({\overline{E}}){\overline{E}} (1-{\overline{E}}) \end{aligned}$$

and if we substitute this into (3.20a) and solve for $G'({\overline{E}})$ we obtain

5.4 $$\begin{aligned} G^{\prime }({\overline{E}})=\frac{\alpha _{1}+\mu +G({\overline{E}})}{{\overline{E}}\left( 1-{\overline{E}}\right) }\frac{1-l\theta }{1-2\theta } \end{aligned}$$

which, by (5.1) and (5.2) is an explicit expression in $\alpha _{1}$, $\alpha _{2}$, $\alpha _{3}$, $\mu$, $\theta$ and the function *G*.

**Fig. 4:**
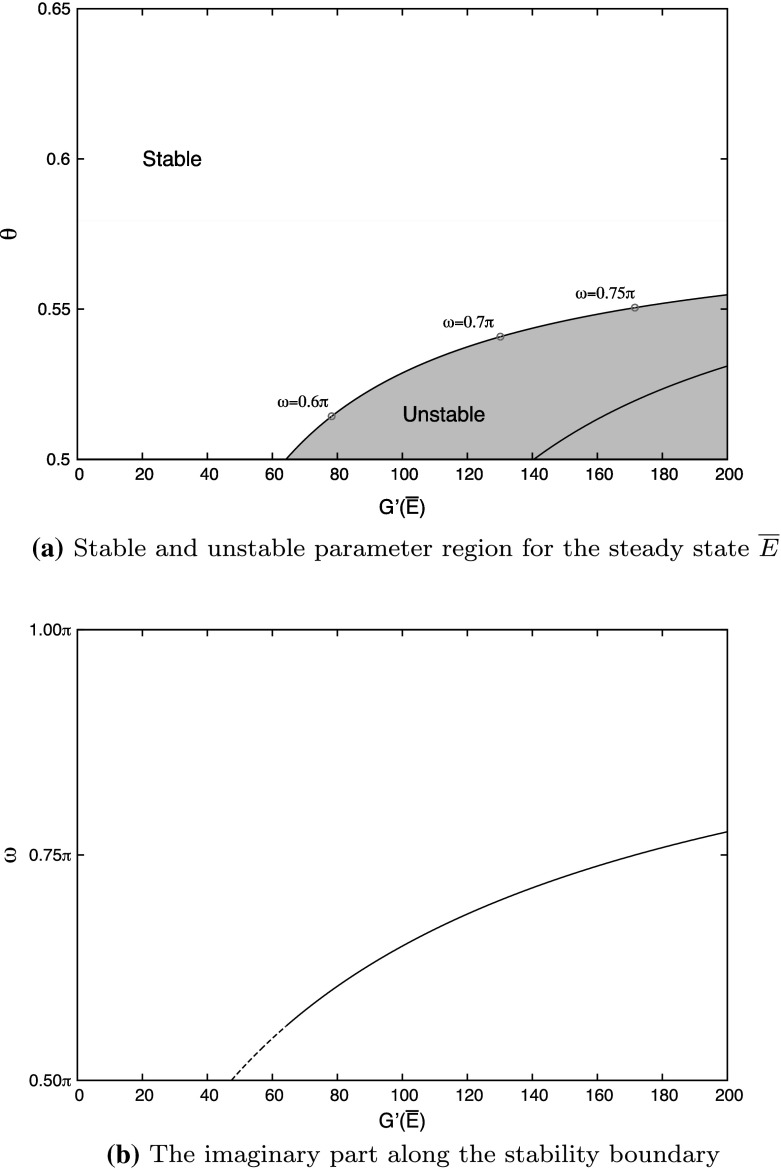
**a** Stable and unstable parameter regions in the $(G^{\prime }({\overline{E}}),\theta )$-plane for the case of regulated duration of quiescence. The parametric curve $C_{0}$ in the $(\alpha _{1},\alpha _{2})$-plane is transformed to the outermost curve in the $(G^{\prime }({\overline{E}}),\theta )$-plane (stability boundary). The curve inside the instability region corresponds to $C_{1}^{+}$. The equilibrium becomes unstable for small $\theta$ and large $G^{\prime }({\overline{E}})$. **b** Graph of the imaginary part $\omega$ along the stability boundary. On the *dashed curve* $\theta <\frac{1}{2}$ while on the *continuous curve* $\theta >\frac{1}{2}$

For given $\beta _{1},\ \mu$ and *G* the expressions (5.3) and (5.4) allow us to transform the stability boundary from $C_{0}$ in the $(\alpha _{1},\alpha _{2})$ plane to the $(G^{\prime }({\overline{E}}),\theta )$ plane. The result, depicted in Fig. 4a for $\mu =0.5,\ \beta _{1}=0.5$ and ${\overline{E}}=0.5$, clearly demonstrates that a steep response promotes instability. Of course $G'({\overline{E}})$ is not really a free parameter and as a consequence Fig. 4 should be regarded as an illustration of a general phenomenon. In order to obtain further biological insights, one would need to consider the dependence on mechanistic parameters that shape the function *G*.

### Regulation via the fraction of cells that become quiescent

In our second case study we assume that *G* is independent of *E*. Recalling (3.6) we can write (3.7) in the form

5.5 $$\begin{aligned} 2e^{-\mu }\beta _{1}({\overline{E}})=1+G\frac{1- 2e^{-\mu }}{\mu }. \end{aligned}$$

Since the right hand side does not depend on ${\overline{E}}$ or $\theta$, we conclude from (3.20c) that once again $\alpha _{3}$ is a constant (now determined by $\mu$ and *G*). Then we get

5.6 $$\begin{aligned} {\overline{E}}=\beta _{1}^{-1}\left( \frac{\alpha _{3}}{2e^{-\mu }} \right) . \end{aligned}$$

Eliminating $A({\overline{E}})$ from (3.20a) and (3.20b) we find exactly as before the expression (5.3) for $\theta$. Solving (3.20a) for $\beta _{1}^{\prime }({\overline{E}})$ we obtain

$$\begin{aligned} \beta _{1}^{\prime }({\overline{E}})=\frac{2e^{-\mu }-1}{2e^{-\mu }\mu }\frac{\alpha _{1}+\mu +G}{{\overline{E}}\left( 1-{\overline{E}}\right) }\frac{1-l\theta }{1-2\theta }, \end{aligned}$$

which only differs from the right hand side of (5.4) by a factor fully determined by $\mu$.

We conclude that, apart from a scaling of the axis, the stability domain is exactly the same as in the situation of Sect. 5.1. In other words, these stability considerations do NOT yield any information that, as we had hoped when embarking on our investigation, helps to decide on the basis of observable fluctuations whether the regulation works via initiation or via termination of quiescence. So it remains a wide open question whether model considerations are at all useful when trying to decide about this issue?

## Discussion

Physiologically structured population models lead to delay equations (Diekmann et al. 2010, this paper). A first step in the subsequent analysis is, as a rule, an investigation of the dependence of steady states, and in particular their stability, on parameters. Thus characteristic equations enter the scene.

Characteristic equations come, in a sense, with their own natural parameters. As emphasized in Diekmann et al. (1991), and more recently echoed in Diekmann and Korvasova (2013), it is attractive to single out two parameters and determine curves in the two parameter plane corresponding to roots lying on the imaginary axis, so to roots being critical, i.e., neither contributing to stability nor to instability. If the characteristic equation has more than two parameters, this leads to two dimensional slices of a higher dimensional parameter space. With a bit of luck one can sometimes understand the full picture in terms of parameterized families of two dimensional sections. Here we have been lucky indeed.

The natural parameters of the characteristic equation are themselves functions of the model parameters. So after one has obtained a picture in terms of the natural parameters, it still remains to analyse how natural parameters change when model parameters change. In Sect. 4 we did exactly this: we studied how $\alpha _{1}$ and $\alpha _{2}$ change when $\theta$ varies between 0 and 1. But an efficient and attractive alternative is to single out TWO model parameters and to map the stability boundary to the corresponding plane of model parameters, as indeed we did in Sect. 5.

Our motivation for studying the characteristic equation (1.3) came from the cell model described in Alarcón et al. (2014) and in Sect. 3. So it was tempting to organise our results differently, in particular to begin with the model, derive the characteristic equation and then study it. Our decision to put, instead, the characteristic equation itself in the spotlight is rooted in the belief that this characteristic equation arises in other contexts and that, once the analysis in terms of natural parameters has been done, other applications only require the study of the map sending model parameters to natural parameters and its inverse. In other words, we hope (and expect) that Sect. 2 is the most useful part of the paper.

Both the initiation of quiescence and the termination of quiescence involve environmental signals. Here we have assumed that one and the same signal is involved, viz. the concentration of an essential resource like oxygen. Consumption of the resource creates a nonlinear feedback loop. Does it matter whether regulation occurs via initiation of quiescence or via termination of quiescence? Is it possible to infer from observed dynamics which of the two mechanisms is the dominant one? Here we have shown that this is not easy, if possible at all. Indeed, we found that destabilization of the steady state hinges on

- little difference in oxygen consumption of proliferating and quiescent cells
- steep response to differences in oxygen concentration near the steady state value

but is rather independent of the precise way in which the feedback acts.

In the present paper we have neither discussed the well-posedness of system (3.3) nor the justification of the Principle of Linearized Stability. Both Alarcón et al. (2014) and Borges et al. (2014) deal with these issues for distributed delay variants of the model. In Alarcón et al. (2014) it is also shown that one can take the limit in the characteristic equation for the delay kernel tending to a Dirac mass and arrive at the characteristic equation considered here. But the limit is not yet considered for the delay equations themselves. In work in progress, S. M. Verduyn Lunel and O. Diekmann are considering duality for neutral equations and this will probably make it unnecessary to consider limits. Concerning the Principle of Linearized Stability, the recent Diekmann and Korvasova (2016) does provide inspiration, but details certainly require attention.

### Electronic supplementary material

Supplementary material 1 (gif 454 KB)

## Appendix: Extinction if $R_{0}(1)<1$ and unbounded growth if $R_{0}(0)>1$

Here we substantiate Remark 3.2. As a preliminary step, we identify the key biologically interpretable quantities that depend on *E* in a monotone manner.

Consider a cell that is quiescent at time *s*. The probability that this cell is reactivated in the time interval [*s*, *t*], with $t>s$, is given by

7.1 $$\begin{aligned} \int _{s}^{t}G(E(\sigma ))e^{-\int _{s}^{\sigma }\left( \mu +G(E(\tau ))\right) d\tau }d\sigma . \end{aligned}$$

By partial integration we see that this is equal to

7.2 $$\begin{aligned} 1-e^{-\mu \left( t-s\right) -\int _{s}^{t}G(E(\tau ))d\tau }- \mu \int _{s}^{t}e^{-\int _{s}^{\sigma }\left( \mu +G(E(\tau ))\right) d\tau }d\sigma , \end{aligned}$$

where the third term is the probability of death and the second the probability that the cell neither died nor was reactivated, so is still quiescent. In self explaining notation we write

7.3 $$\begin{aligned} P_{r}(E(\cdot ))=1-P_{q}(E(\cdot ))-P_{d}(E(\cdot )). \end{aligned}$$

Now suppose that $E_{1}(\tau )\ge E_{2}(\tau )$ for $s\le \tau \le t$. The monotonicity of *G* implies that

$$\begin{aligned} P_{q}(E_{1}(\cdot ))&\le P_{q}(E_{2}(\cdot ))\\ P_{d}(E_{1}(\cdot ))&\le P_{d}(E_{2}(\cdot )) \end{aligned}$$

and hence that

7.4 $$\begin{aligned} P_{r}(E_{1}(\cdot ))\ge P_{r}(E_{2}(\cdot )). \end{aligned}$$

If $E(\tau )$ is constant for $s\le \tau \le t$, taking the value ${\tilde{E}}$, we compute

7.5 $$\begin{aligned} P_{r}({\tilde{E}})=\frac{G({\tilde{E}})}{\mu + G({\tilde{E}})}\left( 1-e^{-\left( \mu +G({\tilde{E}})\right) \left( t-s\right) }\right) . \end{aligned}$$

So for any function *E* defined on [*s*, *t*] with values in [0, 1] (recall (3.3c), repeated as (7.8c) below) we have

7.6 $$\begin{aligned} \frac{G(0)}{\mu +G(0)}\left( 1-e^{-\left( \mu +G(0)\right) \left( t-s\right) }\right)&\le P_{r}(E(\cdot ))\nonumber \\&\le \frac{G(1)}{\mu +G(1)}\left( 1-e^{-\left( \mu +G(1)\right) \left( t-s\right) }\right) \nonumber \\&\le \frac{G(1)}{\mu +G(1)}. \end{aligned}$$

Defining the population birth rate by

7.7 $$\begin{aligned} b(t)=2e^{-\mu }p(t-1), \end{aligned}$$

the model equation (3.3) becomes

7.8a $$\begin{aligned} p(t)&=\beta _{1}(E(t))b(t)+G(E(t))Q(t), \end{aligned}$$

7.8b $$\begin{aligned} \frac{d}{dt}Q(t)&=\beta _{2}(E(t))b(t)-(\mu +G(E(t)))Q(t), \end{aligned}$$

7.8c $$\begin{aligned} E(t)&=\frac{1}{1+\theta \int _{0}^{1}p(t-a)e^{-\mu a}da+\left( 1-\theta \right) Q(t)} \end{aligned}$$

with as state-space $L_{1}[-1,0]\times {\mathbb {R}}$. For a real, not necessarily positive and/or small, number $\epsilon$ we compute

7.9 $$\begin{aligned} \int _{0}^{t+1}b(s)e^{\epsilon s}ds&=2e^{-\mu +\epsilon } \int _{0}^{t+1}p(s-1)e^{\epsilon \left( s-1\right) }ds\nonumber \\&=2e^{-\mu +\epsilon }\left( \int _{0}^{t}p(s)e^{\epsilon s}ds +\int _{-1}^{0}\varphi (s)e^{\epsilon s}ds\right) , \end{aligned}$$

where $\varphi$ is the initial condition for *p*. From (7.8a) one can see that

$$\begin{aligned} \int _{0}^{t}p(s)e^{\epsilon s}ds=\int _{0}^{t} \left( \beta _{1}(E(s))b(s)+G(E(s))Q(s)\right) e^{\epsilon s}ds. \end{aligned}$$

By the variation-of-constants-formula we solve (7.8b) as

$$\begin{aligned} Q(t)=q(t)+\int _{0}^{t}\beta _{2}(E(s))b(s)e^{-\int _{s}^{t} \left( \mu +G(E(\tau ))\right) d\tau }ds, \end{aligned}$$

where $q(t):=Q(0)e^{-\mu t-\int _{0}^{t}G(E(\tau ))d\tau }$. It then holds that

7.10 $$\begin{aligned}&\int _{0}^{t}G(E(s))Q(s)e^{\epsilon s}ds\nonumber \\&\quad = \int _{0}^{t}G(E(s)) q(s)e^{\epsilon s}ds\nonumber \\&\quad \quad +\int _{0}^{t}G(E(s))e^{\epsilon s}\int _{0}^{s}\beta _{2} (E(\theta ))b(\theta )e^{-\int _{\theta }^{s}\left( \mu +G(E(\tau ))\right) d\tau }d\theta ds. \end{aligned}$$

Changing the order of integration, we find

7.11 $$\begin{aligned}&\int _{0}^{t}G(E(s))e^{\epsilon s}\int _{0}^{s}\beta _{2} (E(\theta ))b(\theta )e^{-\int _{\theta }^{s}\left( \mu +G(E(\tau ))\right) d\tau }d\theta ds\nonumber \\&\quad = \int _{0}^{t}\beta _{2}(E(\theta ))b(\theta )e^{\epsilon \theta }\int _{\theta }^{t}G(E(s))e^{-\int _{\theta }^{s}\left( \mu -\epsilon +G(E(\tau ))\right) d\tau }dsd\theta . \end{aligned}$$

Therefore we obtain

7.12 $$\begin{aligned} \int _{0}^{t+1}b(s)e^{\epsilon s}ds=m_{\epsilon }(t)+2e^{-\mu +\epsilon }\int _{0}^{t}b(s)e^{\epsilon s}k(t,s)ds, \end{aligned}$$

where

$$\begin{aligned} m_{\epsilon }(t)&:=2e^{-\mu +\epsilon }\left( \int _{-1}^{0} \varphi (s)e^{\epsilon s}ds+\int _{0}^{t}G(E(s))q(s)e^{\epsilon s}ds\right) ,\\ k(t,s)&:= \beta _{1}(E(s))+\beta _{2}(E(s))\int _{s}^{t}G(E(\theta ))e^{-\int _{s}^{\theta }\left( \mu -\epsilon +G(E(\tau ))\right) d\tau }d\theta . \end{aligned}$$

Note that $t\mapsto m_{\epsilon }(t)$ is monotone increasing and that for $\epsilon <\mu$ this function is bounded. The identity (7.1)$=$(7.2) will be used for the estimation of the kernel *k*.

We first estimate *k*(*t*, *s*) for $\epsilon =0$ from above. As shown in (7.6) we have

$$\begin{aligned} P_{r}(E(\cdot ))=\int _{s}^{t}G(E(\theta ))e^{-\int _{s}^{\theta } \left( \mu +G(E(\tau ))\right) d\tau }d\theta \le \frac{G(1)}{\mu +G(1)}. \end{aligned}$$

Using $\beta _{2}=1-\beta _{1}$ and the monotonicity of $\beta _{1}$, one has, since $\frac{G(1)}{\mu +G(1)}<1$,

$$\begin{aligned} k(t,s) \le \beta _{1}(E(s))+\beta _{2}(E(s))\frac{G(1)}{\mu +G(1)}\le \beta _{1}(1)+\beta _{2}(1)\frac{G(1)}{\mu +G(1)}. \end{aligned}$$

Define

$$\begin{aligned} \eta _{1}(\epsilon ):= 2e^{-\mu +\epsilon }\left( \beta _{1}(1)+ \beta _{2}(1)\frac{G(1)}{\mu +G(1)}\right) =2e^{-\mu +\epsilon }\frac{\mu \beta _{1}(1)+G(1)}{\mu +G(1)}=e^{\epsilon }R_{0}(1). \end{aligned}$$

Now let us assume that $R_{0}(1)<1$. We choose $\epsilon$ such that $0<\epsilon <\mu$ and $\eta _{1}(\epsilon )<1$ hold. Then from (7.12) we obtain

$$\begin{aligned} U(t+1)\le m_{\epsilon }(t)+\eta _{1}(\epsilon )U(t) \end{aligned}$$

with $U(t):= \int _{0}^{t}b(s)e^{\epsilon s}ds$. Thus *U* is bounded. Let us define

7.13 $$\begin{aligned} N(t)=P(t)+Q(t), \end{aligned}$$

where *P* denotes the proliferating cell population given as

7.14 $$\begin{aligned} P(t):= \int _{0}^{1}p(t-a)e^{-\mu a}da. \end{aligned}$$

Now we show that $\lim _{t\rightarrow \infty }N(t)=0$. From (3.3) and the definition of *N* (7.13) one obtains

$$\begin{aligned} \frac{d}{dt}N(t) =p(t)-p(t-1)e^{-\mu }-\mu P(t)+ \beta _{2}(E(t))b(t)-\left( \mu +G(E(t))\right) Q(t). \end{aligned}$$

Using (7.7) and (7.8a), one sees

$$\begin{aligned} \frac{d}{dt}N(t)=\frac{1}{2}b(t)-\mu N(t). \end{aligned}$$

Let us define $N_{\epsilon }(t) := N(t)e^{\epsilon t}$. Then

7.15 $$\begin{aligned} \frac{d}{dt}N_{\epsilon }(t)=\frac{1}{2}b(t)e^{\epsilon t} -(\mu -\epsilon )N_{\epsilon }(t). \end{aligned}$$

Integrating both sides from 0 to *t*, one obtains

7.16 $$\begin{aligned} N_{\epsilon }(t) =N(0)+\frac{1}{2}U(t)-(\mu -\epsilon ) \int _{0}^{t}N_{\epsilon }(s)ds. \end{aligned}$$

Assume that $\int _{0}^{\infty }N_{\epsilon }(s)ds=\infty$. Since *U* is bounded, we obtain $\lim _{t\rightarrow \infty }N_{\epsilon }(t)=-\infty$, which is a contradiction to that *N* is a positive function. Thus $\int _{0}^{\infty }N_{\epsilon }(s)ds<\infty$ follows, which then implies that $N_{\epsilon }$ is bounded because of (7.16). Therefore, we now see that

$$\begin{aligned} \lim _{t\rightarrow \infty }N(t)\le \lim _{t\rightarrow \infty }Me^{-\epsilon t}=0 \end{aligned}$$

for some $M>0$. This implies that $\lim _{t\rightarrow \infty }Q(t)=0$ and that $\lim _{t\rightarrow \infty }P(t)=0$. Note that if the initial condition for *p* has an integrable singularity, this singularity repeats with period 1 and in particular, *p* is not bounded.

Next we assume $R_{0}(0)>1$ holds. Let us define

$$\begin{aligned} \eta _{2}(c_{1},c_{2}):= 2e^{-\mu +c_{1}}\left( \beta _{1}(0)+ \beta _{2}(0)\frac{G(0)}{\mu -c_{1}+G(0)}\left( 1-e^{- \left( \mu -\epsilon +G(0)\right) c_{2}}\right) \right) , \end{aligned}$$

for $c_{1}\le 0$ and $c_{2}>0$. Remark that

$$\begin{aligned} \eta _{2}(0,\infty ):= \lim _{c_{2}\rightarrow \infty }\eta _{2}(0, c_{2})=R_{0}(0)>1. \end{aligned}$$

We choose $\epsilon <0$ and $T>0$ such that $\eta _{2}(\epsilon ,T)>1$ holds. From the variant of (7.6) with $\mu$ replaced by $\mu -\epsilon$

$$\begin{aligned} \int _{s}^{t}G(E(\theta ))e^{-\int _{s}^{\theta } \left( \mu -\epsilon +G(E(\tau ))\right) d\tau }d\theta \ge \frac{G(0)}{\mu -\epsilon +G(0)}\left( 1-e^{-\left( \mu -\epsilon +G(0)\right) (t-s)}\right) . \end{aligned}$$

Thus for $t>T$ and *s* such that $t-s\ge T$ (i.e. $s\le t-T$) it follows

$$\begin{aligned} k(t,s)&\ge \beta _{1}(E(s))+\beta _{2}(E(s))\frac{G(0)}{\mu -\epsilon +G(0)}\left( 1-e^{-\left( \mu -\epsilon +G(0)\right) T}\right) \nonumber \\&\ge \beta _{1}(0)+\beta _{2}(0)\frac{G(0)}{\mu -\epsilon +G(0)}\left( 1-e^{-\left( \mu -\epsilon +G(0)\right) T}\right) \end{aligned}$$

since $\beta _{2}=1-\beta _{1}$ and $\frac{G(0)}{\mu -\epsilon +G(0)}(1-e^{-(\mu -\epsilon + G(0)))T})<1$. Now it can be seen that

$$\begin{aligned} \int _{0}^{t}b(s)e^{\epsilon s}k(t,s)ds&= \int _{0}^{t-T}b(s) e^{\epsilon s}k(t,s)ds+\int _{t-T}^{t}b(s)e^{\epsilon s}k(t,s)ds\nonumber \\&\ge \int _{0}^{t-T}b(s)e^{\epsilon s}ds\Bigg \{\beta _{1}(0)+ \beta _{2}(0)\frac{G(0)}{\mu -\epsilon +G(0)}\nonumber \\&\quad \times \,\left( 1-e^{-\left( \mu -\epsilon +G(0)\right) T}\right) \Big \} +\int _{t-T}^{t}b(s)e^{\epsilon s}k(t,s)ds. \end{aligned}$$

For $U(t)=\int _{0}^{t}b(s)e^{\epsilon s}ds$, from (7.12) we obtain

$$\begin{aligned} U(t+1) \ge n_{\epsilon }(t)+\eta _{2}(\epsilon ,T)U(t-T),\ t>T, \end{aligned}$$

where

$$\begin{aligned} n_{\epsilon }(t)=m_{\epsilon }(t)+2e^{-\mu +\epsilon }\int _{t-T}^{t} b(s)e^{\epsilon s}k(t,s)ds, \end{aligned}$$

thus $U(t)\rightarrow \infty$ as $t\rightarrow \infty$. Now we prove *N* is unbounded. For $N_{\epsilon }(t)=N(t)e^{\epsilon t}$, similar to above computation, we have (7.15). Integrating both sides of (7.15) from 0 to *t*, one obtains the same equation as in (7.16). Suppose that *N* is bounded. Let $\epsilon <0$. Then $\lim _{t\rightarrow \infty }N_{\epsilon }(t)=0$ holds and one can also see that

$$\begin{aligned} (\mu -\epsilon )\int _{0}^{t}N_{\epsilon }(s)ds<(\mu -\epsilon )M \int _{0}^{t}e^{\epsilon s}ds<\infty , \end{aligned}$$

for some $M>0$. Since *U*(*t*) tends to $\infty$ as $t\rightarrow \infty$, the right hand side of (7.16) tends to $\infty$ as $t\rightarrow \infty$ . Thus we obtain a contradiction to the identity in (7.16) and we can conclude that *N* is unbounded.
