# Supplementary figures and images for: On the characteristic equation λ = α1 + (α2 + α3λ)e-λ and its use in the context of a cell population model
Source: J Math Biol. 2015 Aug 6;72:877–908. doi: 10.1007/s00285-015-0918-8 (PMC4751237; doi:10.1007/s00285-015-0918-8)

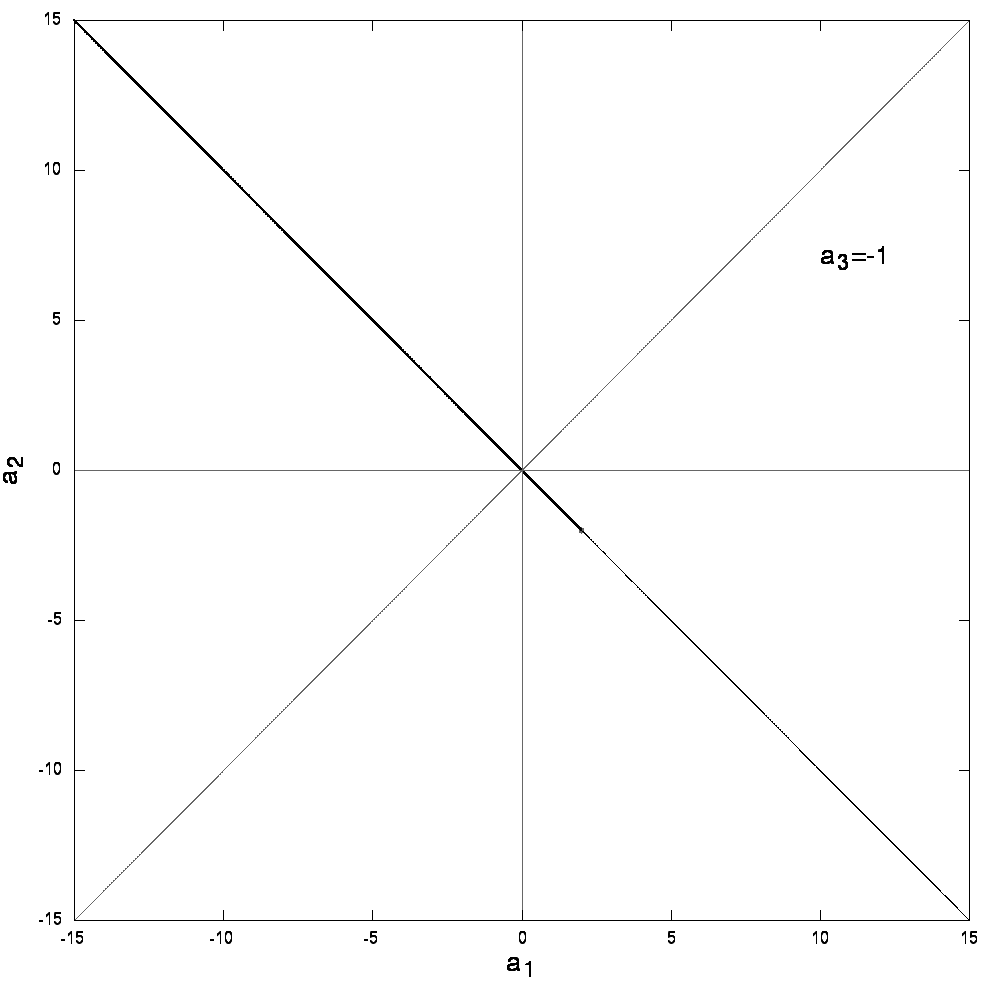

Supplement: Supplementary file 1 — Supplementary material 1 (gif 454 KB) [file 285_2015_918_MOESM1_ESM.gif]
